# Inducing mitochondriopathy-like damages by transformable nucleopeptide nanoparticles for targeted therapy of bladder cancer

**DOI:** 10.1093/nsr/nwae028

**Published:** 2024-01-22

**Authors:** Da-Yong Hou, Ni-Yuan Zhang, Lu Wang, Mei-Yu Lv, Xiang-Peng Li, Peng Zhang, Yue-Ze Wang, Lei Shen, Xiu-Hai Wu, Bo Fu, Peng-Yu Guo, Zi-Qi Wang, Dong-Bing Cheng, Hao Wang, Wanhai Xu

**Affiliations:** NHC and CAMS Key Laboratory of Molecular Probe and Targeted Theranostics, Heilongjiang Key Laboratory of Scientific Research in Urology, Harbin Medical University, Harbin 150001, China; Department of Urology, Harbin Medical University Cancer Hospital, Harbin 150001, China; CAS Key Laboratory for Biomedical Effects of Nanomaterials and Nanosafety, CAS Center for Excellence in Nanoscience, National Center for Nanoscience and Technology (NCNST), Beijing 100190, China; NHC and CAMS Key Laboratory of Molecular Probe and Targeted Theranostics, Heilongjiang Key Laboratory of Scientific Research in Urology, Harbin Medical University, Harbin 150001, China; Department of Urology, Harbin Medical University Cancer Hospital, Harbin 150001, China; NHC and CAMS Key Laboratory of Molecular Probe and Targeted Theranostics, Heilongjiang Key Laboratory of Scientific Research in Urology, Harbin Medical University, Harbin 150001, China; NHC and CAMS Key Laboratory of Molecular Probe and Targeted Theranostics, Heilongjiang Key Laboratory of Scientific Research in Urology, Harbin Medical University, Harbin 150001, China; Department of Urology, Harbin Medical University Cancer Hospital, Harbin 150001, China; NHC and CAMS Key Laboratory of Molecular Probe and Targeted Theranostics, Heilongjiang Key Laboratory of Scientific Research in Urology, Harbin Medical University, Harbin 150001, China; Department of Urology, Harbin Medical University Cancer Hospital, Harbin 150001, China; NHC and CAMS Key Laboratory of Molecular Probe and Targeted Theranostics, Heilongjiang Key Laboratory of Scientific Research in Urology, Harbin Medical University, Harbin 150001, China; Department of Urology, Harbin Medical University Cancer Hospital, Harbin 150001, China; School of Chemistry, Chemical Engineering & Life Science, Hubei Key Laboratory of Nanomedicine for Neurodegenerative Diseases, Wuhan University of Technology, Wuhan 430070, China; NHC and CAMS Key Laboratory of Molecular Probe and Targeted Theranostics, Heilongjiang Key Laboratory of Scientific Research in Urology, Harbin Medical University, Harbin 150001, China; Department of Urology, Harbin Medical University Cancer Hospital, Harbin 150001, China; NHC and CAMS Key Laboratory of Molecular Probe and Targeted Theranostics, Heilongjiang Key Laboratory of Scientific Research in Urology, Harbin Medical University, Harbin 150001, China; Department of Urology, Harbin Medical University Cancer Hospital, Harbin 150001, China; NHC and CAMS Key Laboratory of Molecular Probe and Targeted Theranostics, Heilongjiang Key Laboratory of Scientific Research in Urology, Harbin Medical University, Harbin 150001, China; Department of Urology, Harbin Medical University Cancer Hospital, Harbin 150001, China; NHC and CAMS Key Laboratory of Molecular Probe and Targeted Theranostics, Heilongjiang Key Laboratory of Scientific Research in Urology, Harbin Medical University, Harbin 150001, China; Department of Urology, Harbin Medical University Cancer Hospital, Harbin 150001, China; School of Chemistry, Chemical Engineering & Life Science, Hubei Key Laboratory of Nanomedicine for Neurodegenerative Diseases, Wuhan University of Technology, Wuhan 430070, China; CAS Key Laboratory for Biomedical Effects of Nanomaterials and Nanosafety, CAS Center for Excellence in Nanoscience, National Center for Nanoscience and Technology (NCNST), Beijing 100190, China; NHC and CAMS Key Laboratory of Molecular Probe and Targeted Theranostics, Heilongjiang Key Laboratory of Scientific Research in Urology, Harbin Medical University, Harbin 150001, China; Department of Urology, Harbin Medical University Cancer Hospital, Harbin 150001, China

**Keywords:** self-assembly, nucleopeptide, nanomaterial, bladder cancer, mitochondriopathy

## Abstract

Mitochondriopathy inspired adenosine triphosphate (ATP) depletions have been recognized as a powerful way for controlling tumor growth. Nevertheless, selective sequestration or exhaustion of ATP under complex biological environments remains a prodigious challenge. Harnessing the advantages of *in vivo* self-assembled nanomaterials, we designed an Intracellular ATP Sequestration (IAS) system to specifically construct nanofibrous nanostructures on the surface of tumor nuclei with exposed ATP binding sites, leading to highly efficient suppression of bladder cancer by induction of mitochondriopathy-like damages. Briefly, the reported transformable nucleopeptide (NLS-FF-T) self-assembled into nuclear-targeted nanoparticles with ATP binding sites encapsulated inside under aqueous conditions. By interaction with KPNA2, the NLS-FF-T transformed into a nanofibrous-based ATP trapper on the surface of tumor nuclei, which prevented the production of intracellular energy. As a result, multiple bladder tumor cell lines (T24, EJ and RT-112) revealed that the half-maximal inhibitory concentration (IC50) of NLS-FF-T was reduced by approximately 4-fold when compared to NLS-T. Following intravenous administration, NLS-FF-T was found to be dose-dependently accumulated at the tumor site of T24 xenograft mice. More significantly, this IAS system exhibited an extremely antitumor efficacy according to the deterioration of T24 tumors and simultaneously prolonged the overall survival of T24 orthotopic xenograft mice. Together, our findings clearly demonstrated the therapeutic advantages of intracellular ATP sequestration-induced mitochondriopathy-like damages, which provides a potential treatment strategy for malignancies.

## Teaser

An Intracellular ATP Sequestration (IAS) system was designed for targeted therapy of bladder cancer by induction of mitochondriopathy-like damages.

## INTRODUCTION

Around 75% of bladder cancer cases are classified as non-muscle invasive bladder cancer (NMIBC) and the primary treatment methods used are transurethral resection of bladder tumor (TURBT) and chemotherapy [1–3]. At present, first line drugs such as mitomycin C, epirubicin, pirarubicin, gemcitabine and Bacillus Calmette-Guér (BCG) utilized for intravesical treatment of bladder cancer have revealed a beneficial effect [4,5]. Nevertheless, mounting evidence has demonstrated that postoperative administration with drugs has shown failure of treatment owing to the low drug activity against more aggressive tumors and severe side-effects [6,7]. Furthermore, NMIBC still presents a recurrence rate of 50% to 70% even after TURBT and immediate treatment with chemotherapeutic drugs, making bladder cancer one of the solid tumors most expensive to treat [8,9]. Consequently, development of novel drugs against tumors, with minimized systemic toxicity, may substantially improve the treatment outcomes of NMIBC patients [10].

By altering metabolic pathways, tumor cells exhibit heightened energy production and biosynthetic processes to fuel the uncontrolled growth [11]. Accordingly, adenosine triphosphate (ATP) is reported to be upregulated and excessively concentrated in tumor cells, which plays a central role in various intracellular processes, including cellular respiration, signaling transduction and enzyme catalysis [12,13]. For several decades, targeted regulation of the metabolic pathways has been considered a promising approach for tumor therapy [14,15]. However, few clinical trials bring enough benefits for patients using a tumor nutrient starvation strategy. In contrast to the heightened ATP production of tumor cells, mitochondriopathy refers to a group of genetic disorders which affect the function of mitochondria, the organelles responsible for energy production. Owing to the decreased ATP synthesis and insufficient energy production, a wide range of symptoms, including muscle weakness, neurological problems and organ failure will arise spontaneously. Therefore, mitochondriopathy inspired ATP depletions have been widely recognized as a powerful way for controlling tumor growth via reducing intracellular energy production [16,17]. Nevertheless, selective sequestration or exhaustion of ATP under complex biological environments remain a prodigious challenge [18]. Currently, supramolecular chemistry has been actively developed for tumor diagnosis and therapy with favorable biocompatibility and high tumor specificity [19–22]. Therefore, the *in vivo* self-assembly based nanomaterials have been extensively investigated in the development of anticancer agents providing improved subcellular accumulation and tumor therapeutic efficacy by integrating multiple moieties [23,24].

**Figure 1.**
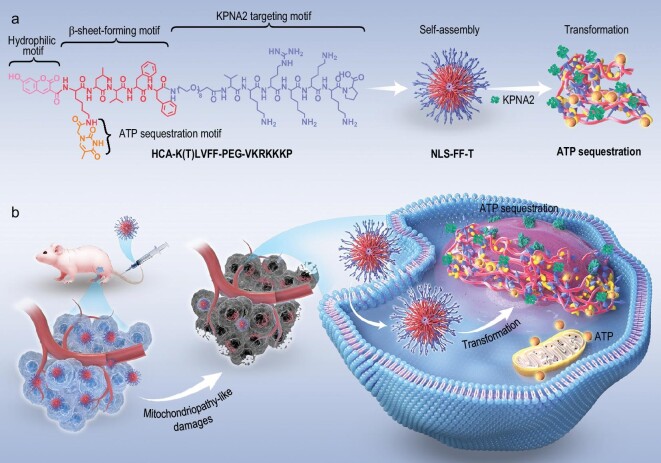
Inducing mitochondriopathy-like damages by transformable nucleopeptide nanoparticles for targeted therapy of bladder cancer. (a) This designed and synthesized NLS-FF-T (HCA-K(T)LVFF-PEG-VKRKKKP) peptide was comprised of five discrete functional motifs: (i) coumarin (HCA), as a hydrophobic core to induce the formation of nanoparticles; (ii) thymine, as ATP selective sequestering moiety; (iii) KLVFF, β-sheet-forming peptide domain derived from β-amyloid (Aβ) peptide; (iv) PEG, as a hydrophilic surface to induce the formation of nanoparticles and (v) the VKRKKKP, KPNA2-binding peptide. (b) Briefly, the reported transformable nucleopeptide (NLS-FF-T) self-assembled into nuclear-targeted nanoparticles with ATP binding sites encapsuled inside under aqueous conditions. By interaction with KPNA2, the NLS-FF-T transformed into nanofibrous-based ATP trapper on the surface of tumor nuclear, which prevented the production of intracellular energy.

Utilizing *in vivo* self-assembled nanomaterials [25–27], we designed an Intracellular ATP Sequestration (IAS) system for targeted suppression of bladder cancer. The IAS system involves constructing nanofibrous nanostructures on the surface of tumor nuclei with exposed ATP binding sites, inducing mitochondriopathy-like damages (Fig. 1). Our transformable nucleopeptide (NLS-FF-T) self-assembles into nuclear-targeted nanoparticles with encapsulated ATP sites. Upon interaction with karyopherin subunit alpha-2 (KPNA2), NLS-FF-T transforms into nanofibrous-based ATP trappers, impeding intracellular energy production. In our study, multiple bladder tumor cell lines (T24, EJ and RT-112) exhibited approximately a 4-fold decrease in the half-maximal inhibitory concentration (IC50) for NLS-FF-T compared to NLS-T. Intravenous administration of an IAS system resulted in dose-dependent accumulation at the tumor site in T24 xenograft mice. Notably, the IAS system demonstrated remarkable antitumor efficacy, as evidenced by the degradation of T24 tumors, and an extended overall survival of T24 orthotopic xenograft mice. Our findings highlight the therapeutic advantages of intracellular ATP sequestration-induced mitochondriopathy-like damages, suggesting a potential treatment strategy for malignancies.

## RESULTS AND DISCUSSION

### KPNA2-mediated transformation and ATP sequestration of the IAS system

**Figure 2.**
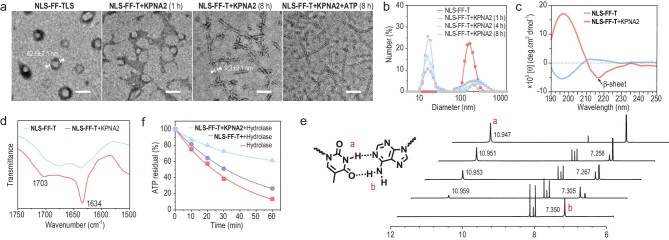
KPNA2-mediated transformation and ATP sequestration of the IAS system. (a) Representative TEM images of NLS-FF-T, NLS-FF-T + KPNA2 at different time points (1 and 8 h) and NLS-FF-T + KPNA2 + ATP (200 μM) (8 h). Scale bar: 100 nm. (b) Variation in size distribution of initial NLS-FF-T and NLS-FF-T interaction with KPNA2 at different time points. (c) Time-dependent CD spectra of NLS-FF-T (50 μM) after interaction with KPNA2 in deionized water. (d) FTIR spectra of NLS-FF-T (50 μM) and NLS-FF-T interaction with KPNA2 in deionized water. (e) ^1^H-NMR (400 MHz, CDCl_3_/DMSO-*d_6_* = 1/1) spectra of NLS-FF-T blended with various ratios of ATP (from bottom to top, thymine to ATP (T/ATP) molar ratios: 0:1, 3:7, 5:5, 7:3 and 1:0). (f) ATP hydrolysis profiles of NLS-FF-T nanoparticles and nanofibers measured by bioluminescence after the treatment of alkaline phosphatase (0.5 U/mL).

This designed and synthesized NLS-FF-T (HCA-K(T)LVFF-PEG-VKRKKKP) peptide was comprised of five discrete functional motifs: (i) coumarin (HCA), as a hydrophobic core to induce the formation of nanoparticles; (ii) thymine, as ATP selective sequestering moiety [18]; (iii) KLVFF, β-sheet-forming peptide domain derived from β-amyloid (Aβ) peptide [27–29]; (iv) PEG, as a hydrophilic surface to induce the formation of nanoparticles and (v) the VKRKKKP peptide Karyopherin Subunit Alpha-2 (KPNA2)-binding domain, derived from the primary sequence of NLS (Figs S1 and S2) [30,31]. NLS-FF (HCA-KLVFF-PEG-VKRKKKP), FF-T (HCA-K(T)LVFF-PEG2) and NLS-T (HCA-K(T)LV-PEG-VKRKKKP) were designed and synthesized as controls (Figs S3–S8). The critical aggregation concentration (CAC) of NLS-FF, FF-T, NLS-T and NLS-FF-T was investigated by pyrene probe, which was calculated to be 14.8, 18.2, 14.2 and 11.6 μg/mL, respectively (Fig. S9). Afterward, transmission electron microscopy (TEM) and dynamic light scattering (DLS) were performed to confirm the structural transformation of the IAS system from nanoparticle to nanofibrous (Fig. 2a and b). According to analysis of the TEM observation, NLS-FF-T dispersed as a spherical nanoparticle with a diameter of ∼62.5 ± 7.1 nm, which gradually disintegrated and transformed into nanofibers after interaction with KPNA2 protein (Fig. 2a). When the interaction time increased to 8 h, an obvious nanofibrous structure with diameter of 9.3 ± 2.1 nm was detected with or without ATP. In contrast, no morphology transformation was observed in NLS-FF-T after 24 hours without KPNA2 incubation (Fig. S10). The particle sizes of NLS-FF, FF-T and NLS-T were found to be ∼73 ± 5.2, ∼64 ± 6.6 and ∼56 ± 8.5 nm, respectively, which is like that of NLS-FF-T nanoparticles (Figs S11–S13). The structural transformation of NLS-FF-T was further demonstrated with DLS (Fig. 2b). On the contrary, FF-T and NLS-T incubated with KPNA2 exhibited no changes over 24 hours (Figs S11–S13).

Subsequently, the secondary structure transformation of the IAS system was evaluated with circular dichroism (CD) spectroscopy and Fourier transform infrared spectroscopy (FTIR) [32]. As can be seen in Fig. 2c, the secondary structure of NLS-FF-T was first investigated by CD spectroscopy, which exhibited an increased signal of negative peak at 216 nm and positive peak at 195 nm, indicating the β-sheet formation of NLS-FF-T after interaction with KPNA2 (Fig. 2c). Meanwhile, the intensity of the amide I band at 1634 cm^−1^ obviously increased and the higher energy but weaker shoulder band at 1703 cm^−1^ in the FTIR spectrum further suggested the antiparallel β-sheet formation of NLS-FF-T after interaction with KPNA2 (Fig. 2d).

The molecular interactions between thymine and ATP were analyzed by ^1^H NMR spectra. As shown in Fig. 2e, the addition of thymine to ATP (T/ATP molar ratios: 0 : 1, 3 : 7, 5 : 5, 7 : 3 and 1 : 0) led to high field shift of the signal (N-H) of the thymine group from 10.959 to 10.947 ppm (a). Similarly, the NH_2_ resonance of ATP kept shifting to the high field (b) with the increase in the concentration of added thymine, which appeared at 7.350–7.258 ppm (Fig. 2e). Both the ^1^H NMR signal change of thymine and ATP demonstrated the formation of complementary hydrogen-bonding, which implied the NLS-FF-T could combine and capture ATP. Afterward, the ATP sequestration ability was evaluated depending on the ATP stability in an alkaline phosphatase (ALP) solution (Fig. 2f). As can be seen, the hydrolytic activity of APT after treatment with NLS-FF-T nanofibers was inhibited, and 61% of ATP still remained after ALP catalytic hydrolysis, while only 13% and 27% of ATP was reserved in the blank control and nanoparticle group. The NLS-FF-T nanofibers could efficienly capture and stablize the ATP molecules, which presented the potential to act as an effective system to induce mitochondriopathy-like damages.

### *In vitro* ATP sequestration-based antitumor effect of the IAS system

**Figure 3.**
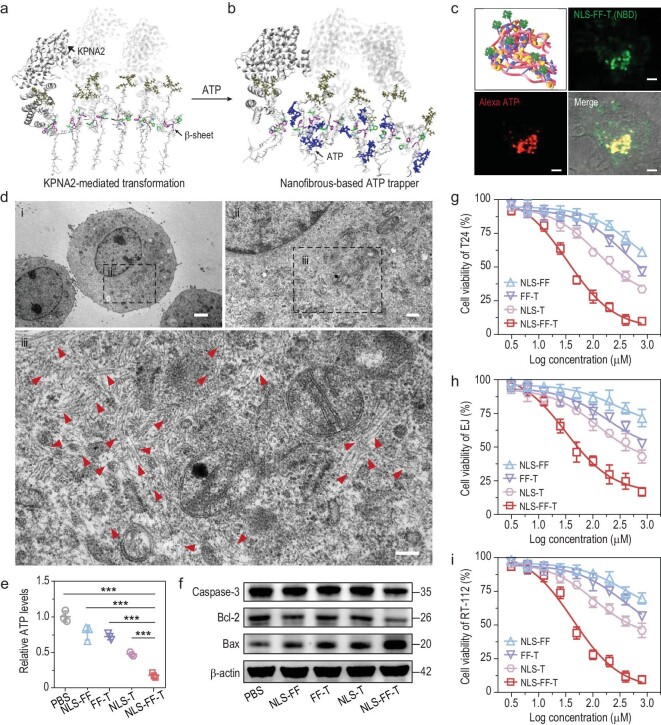
*In vitro* ATP sequestration-based antitumor effect of the IAS system. (a) Molecular dynamics simulation structure of β-sheet-KPNA2 complex. (b) Molecular dynamics simulation structure of β-sheet-KPNA2-ATP complex. The sidechains of the LVFF were highlighted with green and purple. The sidechain of the KRKKK part was highlighted with tan color. The ATP molecule was highlighted with blue color. All structures were displayed by VMD. (c) Schematic illustration of the KPNA2-mediated transformation and ATP sequestration of NLS-FF-T and confocal laser scanning microscopy (CLSM) images of T24 cells incubated with Alexa-ATP and NBD labeled NLS-FF-T (50 μM). Scale bar: 5 μm. (d) Bio-TEM images of T24 cells after treatment with NLS-FF-T (50 μM). The red arrows indicate the intracellular nanofibrils. Scale bars: 2 μm (i), 500 nm (ii) and 100 nm (iii). (e) Intracellular ATP levels of T24 cells after treatment with PBS, NLS-FF, FF-T, NLS-T and NLS-FF-T (50 μM). (f) Western blot analysis of Caspase-3, Bcl-2 and Bax pathways in T24 cells treated with NLS-FF, FF-T, NLS-T and NLS-FF-T for 48 hours. (g) Viability of T24 cells after treatment with NLS-FF, FF-T, NLS-T and NLS-FF-T at different concentrations for 48 hours. (h) Viability of EJ cells after different treatment for 48 hours. (i) Viability of RT-112 cells after different treatment for 48 hours. *P* values were performed with one-way ANOVA followed by *post hoc* Tukey's test. ***P < 0.001. Data were presented as mean ± SD.

The NLS-FF-T was designed to be transformed from nanoparticles into nanofibrous structures by KPNA2 binding with multivalent effect to ATP (Fig. 3a). According to the results of the molecular dynamics simulation for the β-sheet-KPNA2 and the β-sheet-KPNA2-ATP complex systems, we found that the β-sheet has been self-assembled via π-π interactions between Phe5 and Phe6. The interesting thing was that the C-terminal region of the β-sheet, which was rich in positively charged residues (KRKKK), would form a stable electrostatic interaction with KPNA2 (Fig. 3b). When the ATP molecules were docked to Y2 residue, the C-terminal of the β-sheet without KPNA2 binding would be bent and moved closer to the ATP molecule (highly negative charged phosphate group) (Fig. 3b). However, for the β-sheet with the C-terminal stabilized by KPNA2, the structure was quite robust (Fig. 3b). Moreover, the computational diameter of NLS-FF-T nanofiber is ∼9 nm according to the molecular arrangement mode (Fig. S14), which was in accordance with the TEM result. Taken together, these results further confirmed the KPNA2-mediated transformation and ATP sequestration of the IAS system.

Moreover, the lysosomal escape characteristics of NLS-FF-T was investigated by Confocal Laser Scanning Microscopy (CLSM). As a result, the red fluorescence signal of NLS-FF-T was clearly observed outside the lysosomes (stained with LysoTracker green) after being incubated with T24 cells for 4 h, indicating the lysosomes-escape of NLS-FF-T (Fig. S15). To validate the intracellular interaction between transformed NLS-FF-T and ATP (Fig. 3c), NBD-labelled NLS-FF-T was synthesized for fluorescence imaging. As can be seen in Fig. 3c, ATP was observed to be overlapped with NBD-labelled NLS-FF-T according to the representative CLSM images. The results above further confirmed that the intracellular ATP was selectively sequestered in the nanofibrous structures of NLS-FF-T. Meanwhile, no obvious nanostructures were observed in the representative bio-TEM images of PBS treated T24 cells (Fig. S16), while plentiful bundles of nanofibers were observed in the NLS-FF-T treated cells (Fig. 3d), suggesting the intracellular KPNA2-mediated transformation of NLS-FF-T (Fig. 3e). Moreover, the intracellular ATP levels had significantly declined in NLS-FF-T treated T24 cells compared with that in NLS-FF, FF-T and NLS-T treated cells. Furthermore, as symbols of apoptosis, caspase 3, Bcl-2 and Bcl-2-associated X protein (Bax) expression were evaluated by Western blot assay. Compared with the substantial activation of apoptosis in NLS-FF-T treated T24 cells, no apoptosis biomarker was observed in T24 cells under the treatment of NLS-FF, FF-T and NLS-T (Fig. 3f). Subsequently, a CCK-8 assay was employed to evaluate the cytotoxicity of the IAS system in T24, EJ, and RT-112 human bladder cancer cell lines (Fig. 3g–i). The findings reveal that NLS-FF-T demonstrated significantly higher cytotoxicity against T24, EJ, and RT-112 cells with half maximal inhibitory concentration (IC50) values of 34.0, 32.0, and 43.3 nM, respectively. By contrast, NLS-FF (IC50: 531.6, 277.9 and 376.0 nM, respectively), FF-T (IC50: 408.0, 181.3 and 356.4 nM, respectively) and NLS-T (IC50: 119.0, 122.3 and 106.9 nM, respectively) exhibited an apparently declined cytotoxicity against T24, EJ and RT-112 cells (Fig. 3g–i). Toxicity assays of the IAS system towards SV-HUC-1 cells (normal bladder cells) were performed with T-ATP (pure ATP sequestration motif) as control. As a result, the IAS system exhibited an enhanced toxicity towards tumor cells (T24) than that of normal cells (SV-HUC-1), indicating good biocompatibility of NLS-FF-T (Fig. S17a). Meanwhile, the NLS-FF-T demonstrated significantly higher cytotoxicity against T24 cells than that of T-ATP, indicating the enhanced antitumor efficacy of NLS-FF-T by nanofibrous-based ATP sequester (Fig. S17b). Altogether, these results demonstrated that NLS-FF-T nanoparticles transformed into nanofibrous structures by KPNA2 binding, which realized substantial tumor cytotoxicity by intracellular ATP sequestration-induced mitochondriopathy-like damages.

### *In vivo* ATP sequestration-based antitumor effect of the IAS system

**Figure 4.**
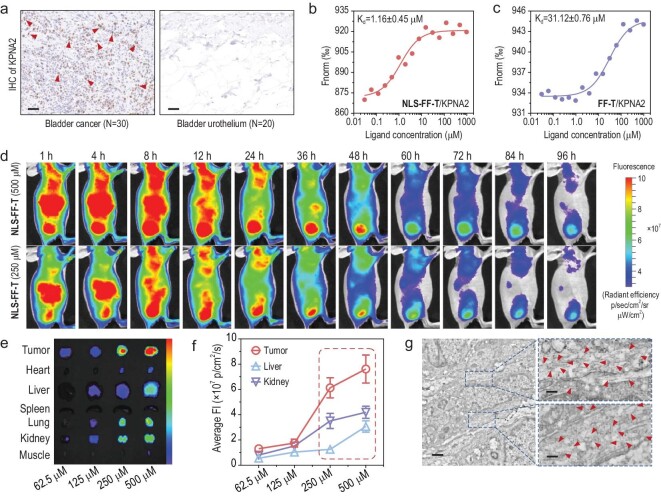
*In vivo* dose-dependent tumor targeting effect of the IAS system. (a) KPNA2 immunohistochemical staining of bladder cancer tissues and bladder urothelium tissues. Scale bars: 50 μm. (b) The binding affinity of NLS-FF-T to KPNA2 by microscale thermophoresis (MST) ligand binding measurements. K_d_: the dissociation constant. (c) The binding affinity of FF-T to KPNA2 by MST ligand binding measurements. (d) Representative *in vivo* fluorescence images of T24 xenograft mice collected after intravenous administration of NLS-FF-T (500 μM, 250 μM, 125 μM or 62.5 μM in 200 μL PBS) (*N* = 3). (e) Dose-dependent *ex vivo* fluorescence images of tumor and major organs after intravenous administration of NLS-FF-T (500 μM, 250 μM, 125 μM or 62.5 μM in 200 μL PBS) (*N* = 3). (f) Corresponding quantitative fluorescence intensity of tumor, liver and kidney after intravenous administration of NLS-FF-T (*N* = 3). (g) Bio-TEM images of T24 tumor tissues after treatment with NLS-FF-T (500 μM in 200 μL PBS). The red arrows indicate the intracellular nanofibrils. Scale bars: 1 μm and 200 nm (zoom in).

As a major nucleocytoplasmic transporter, KPNA2 has been identified to be aberrantly overexpressed in a variety of tumors [33,34]. Consequently, immunohistochemistry (IHC) stain assay was conducted to investigate the expression of KPNA2 in bladder tumor tissues from 30 patients combined with normal bladder tissues from 20 patients. Taken together, the results above suggested that KPNA2 was highly overexpressed in bladder tumor tissues compared with that in normal tissues (Fig. 4a and Fig. S18), which holds great potential in developing tumor specific biomaterials against bladder cancer. Afterward, Microscale Thermophoresis (MST) ligand binding assay was conducted to evaluate the binding affinity of NLS-FF-T and FF-T to KPNA2. As expected, NLS-FF-T exhibited a higher binding affinity to KPNA2 (apparent Kd: ∼1.16 μM) by multivalent cooperative interactions (Fig. 4b). Nevertheless, FF-T showed an extremely decreased binding affinity to KPNA2 (apparent Kd: ∼31.12 μM) owing to the missing motif of KPNA2 targeting peptide (Fig. 4c).

Moreover, the biostability and pharmacokinetics of NLS-FF-T nucleopeptide was investigated. As a result, the NLS-FF-T-KPNA2 exhibited a great colloidal stability (size changes) against serum proteins (e.g. with FBS) for 24 hours (Figs S19 and S20). Subsequently, sequential fluorescence images of T24 xenograft mice were collected after intravenous administration of NLS-FF-T at varying dosing levels (500 μM or 250 μM in 200 μL PBS) for 1, 4, 8, 12, 24, 36, 48, 60, 72, 84 and 96 hours (Fig. 4d). As a result, the NLS-FF-T treated T24 xenograft mice exhibited an apparent dose-dependent tumor accumulation and long-time tumor retention which might be attributed to the *in situ* KPNA2-mediated transformation of NLS-FF-T into nanofibrous structures. To further investigate the dose-dependent biodistribution and metabolism of the IAS system, T24 xenograft mice were intravenously administered NLS-FF-T at a dose of 500 μM, 250 μM, 125 μM or 62.5 μM in 200 μL PBS. Afterward, the tumors and main organs were resected for *ex vivo* fluorescence imaging (Fig. 4e). As a result, the fluorescence signal was detected to be linear enhancement with the increase of dosage in liver and kidney, indicating that NLS-FF-T was predominantly metabolized via liver and kidney (Fig. 4f). On the contrary, substantial fluorescence signals were observed to be exponentially increased on the tumors with increasing dosage (Fig. 4f). Meanwhile, abundant bundles of nanofibers were notable according to the TEM images of excised tumor sections at 12 hours post-injection (Fig. 4g). By contrast, there was an absence of nanostructures in tumor sections that were treated with PBS (Fig. S21), indicating the *in situ* KPNA2-mediated transformation of NLS-FF-T.

### *In vivo* antitumor evaluation of the IAS system in the T24 xenograft model

**Figure 5.**
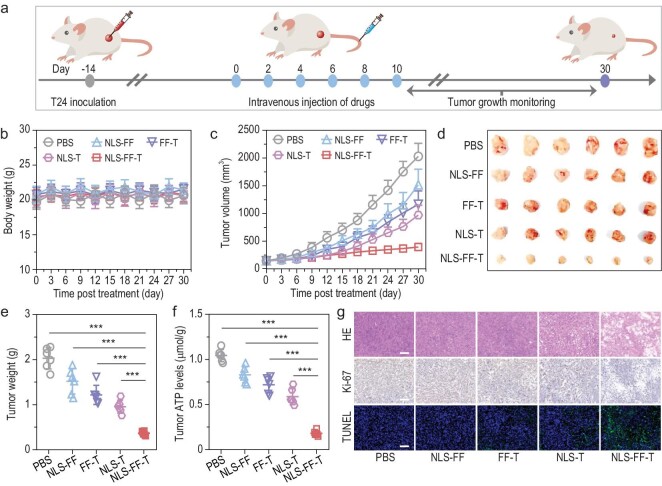
*In vivo* antitumor effect of the IAS system in T24 xenograft model. (a) Schematic illustration of the treatment experiment design. T24 xenograft mice (approximately 6–8 weeks, 16–18 g) were intravenously administered with PBS, NLS-FF, FF-T, NLS-T and NLS-FF-T (500 μM in 200 μL PBS) every 2 days for six times. (b) Body weights of T24 xenograft mice after treatment with PBS, NLS-FF, FF-T, NLS-T and NLS-FF-T (500 μM in 200 μL PBS). (c) Average tumor growth curves of T24 xenograft mice after treatment with PBS, NLS-FF, FF-T, NLS-T and NLS-FF-T (500 μM in 200 μL PBS). (d and e) Brightened field images and tumor weights of harvested T24 tumor tissues after treatment with PBS, NLS-FF, FF-T, NLS-T and NLS-FF-T (500 μM in 200 μL PBS). (f) ATP levels of harvested T24 tumor tissues after treatment with PBS, NLS-FF, FF-T, NLS-T and NLS-FF-T (500 μM in 200 μL PBS). (g) Hematoxylin and eosin (H&E), Ki-67 and terminal deoxynucleotidyl transferase deoxyuridine triphosphate (dUTP) nick end labeling (TUNEL) staining of harvested T24 tumor tissues after treatment with PBS, NLS-FF, FF-T, NLS-T and NLS-FF-T (500 μM in 200 μL PBS). *P* values were performed with one-way ANOVA followed by *post hoc* Tukey's test. ***P < 0.001. Data were presented as mean ± SD.

Considering the promising *in vitro* cytotoxicity results and *in vivo* tumor-targeting ability, the *in vivo* antitumor effect of the IAS system was further evaluated. When the average tumor size reached ∼100–200 mm^3^, T24 xenograft mice were randomly divided into five groups (*N* = 6 mice per group) accompanied by six-time intravenous administration of PBS, NLS-FF, FF-T, NLS-T and NLS-FF-T (500 μM in 200 μL PBS) at 2-day intervals (Fig. 5a). As a result, the T24 xenograft mice treated with NLS-FF-T exhibited no substantial loss of body weight, indicating that NLS-FF-T hold the advantage of low systemic toxicity owing to the encapsuled ATP binding site inside nanoparticles (Fig. 5b). Additionally, the PBS and NLS-FF treated T24 xenograft mice grew exponentially over time with a mean tumor volume of 2030 ± 235 mm^3^ and 1517 ± 283 mm^3^ at 30 days post-injection (Fig. 5c and Fig. S22). For the meantime, the FF-T and NLS-T treated T24 xenograft mice showed moderate antitumor efficacy with an average tumor volume of 1166 ± 243 mm^3^ and 972 ± 176 mm^3^ at 30 days post-injection (Fig. 5c and Fig. S22). Excitingly, the tumors of T24 xenograft mice treated with NLS-FF-T displayed a remarkable antitumor efficacy with an average volume of 397 ± 65 mm^3^ at 30 days post-injection, suggesting the enhanced antitumor efficacy of NLS-FF-T by nanofibrous-based ATP sequester (Fig. 5c and Fig. S22). Besides, the individual variation of tumor growth in T24 xenograft mice within each group was nearly negligible (Fig. S22). Furthermore, the tumor growth inhibition (TGI) rate in NLS-FF-T treated T24 xenograft mice was substantially augmented to 82% compared with that in NLS-FF (25%), FF-T (40%) and NLS-T (53%) treated mice at 30 days post-injection (Fig. 5d and e). Additionally, the ATP levels in tumors correlated well with tumor growth results that NLS-FF-T treated mice exhibited an obviously decreased ATP content compared with PBS, in NLS-FF, FF-T and NLS-T treated mice (Fig. 5f). Moreover, the antitumor effect of NLS-FF-T was further confirmed according to the apparently damaged nuclei and cytoplasm in tumor hematoxylin and eosin (H&E) staining, augmented apoptosis signals in tumor terminal deoxynucleotidyl transferase deoxyuridine triphosphate (dUTP) nick end labeling (TUNEL) staining and declined proliferation signals in tumor Ki67 immunohistochemical (IHC) staining (Fig. 5g). Besides, we have included a positive control (SN-38) and T-ATP (pure ATP sequestration motif) for inhibiting T24 xenograft mice. As a result, the PBS and T-ATP (pure ATP sequestration motif) treated T24 xenograft mice grew exponentially over time at 24 days post-injection (Fig. S23a). In the meantime, the SN38 treated T24 xenograft mice showed moderate antitumor efficacy (Fig. S23a). Excitingly, the tumors of T24 xenograft mice treated with NLS-FF-T displayed a remarkable antitumor efficacy, suggesting the enhanced antitumor efficacy of NLS-FF-T by nanofibrous-based ATP sequester (Fig. S23a). Besides, T24 xenograft mice demonstrated no impact on body weight (Fig. S23b). Taken together, these results demonstrated that the IAS system presented a brilliant antitumor efficacy through induction of mitochondriopathy-like damages and low systemic toxicity, which hold great potential in bladder cancer therapy.

### *In vivo* antitumor recurrence efficacy of the IAS system

**Figure 6.**
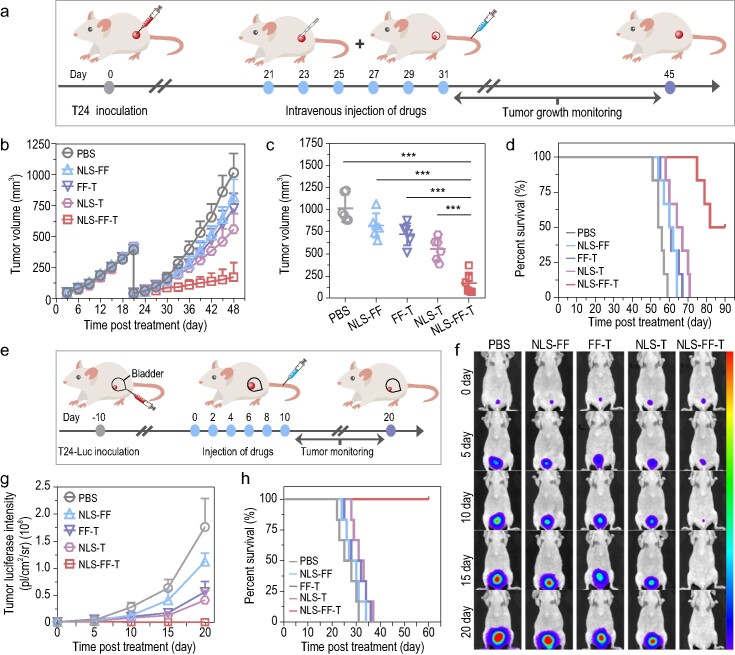
*In vivo* antitumor effect of the IAS system in T24 tumor-recurrence model and orthotopic bladder cancer model. (a) Schematic illustration of the treatment experiment design: T24 xenograft mice (approximately 6–8 weeks, 16–18 g) were treated with PBS, NLS-FF, FF-T, NLS-T and NLS-FF-T (500 μM in 200 μL PBS) every 2 days for six times. (b) Average tumor growth curves of T24 xenograft mice after different treatments. (c) Average tumor volume of T24 xenograft mice after different treatments. (d) Kaplan–Meier survival curve of T24 xenograft mice after administration of different treatments. (e) Schematic illustration of the treatment experiment design: T24 orthotopic bladder cancer mice (approximately 6–8 weeks, 16–18 g) were treated with PBS, NLS-FF, FF-T, NLS-T and NLS-FF-T (500 μM in 200 μL PBS) every 2 days for six times. (f) *In vivo* bioluminescence images of T24 orthotopic bladder cancer mice after different treatments. (g) Bioluminescence intensity at tumor site of T24 orthotopic bladder cancer mice after different treatments. (h) Kaplan–Meier survival curve of T24 orthotopic bladder cancer mice after different treatments. *P* values were performed with one-way ANOVA followed by *post hoc* Tukey's test. ***P < 0.001. Data were presented as mean ± SD.

Animal tumor models that closely mimic the human condition are essential for the preclinical assessment of experimental therapeutics. Therefore, an incomplete tumor resection mouse model was established to investigate the antitumor effects of the IAS system in controlling the regrowth of residual tumors, which is a significant contributor to the high recurrence rate in clinical practice [35]. BALB/c nude mice (approximately 6–8 weeks, 16–18 g) were subcutaneously inoculated with 5 × 10^6^ T24 cells and randomly divided into five groups (with *N* = 6 mice per group). Afterward, most of the tumor tissues were removed, leaving behind roughly 40 mm^3^ tumors, to simulate the residual tumors found in surgery after the average tumor volume reached about 400–500 mm^3^ (Fig. 6a). The T24 xenograft mice were then treated intravenously with PBS, NLS-FF, FF-T, NLS-T or NLS-FF-T (500 μM in 200 μL PBS) once every 2 days for six times. As a result, T24 xenograft mice treated with NLS-FF-T demonstrated significantly improved antitumor efficacy in controlling tumor regrowth, with an average tumor volume of 171 ± 118 mm^3^ at 48 days post-treatment, compared to the PBS treated mice (1018 ± 155 mm^3^) (Fig. 6b and Fig. S24). However, the NLS-FF, FF-T, and NLS-T treated T24 xenograft mice exhibited decreased control of tumor regrowth, with an average tumor volume of 824 ± 138 mm^3^, 725 ± 125 mm^3^ and 558 ± 128 mm^3^, respectively, at 48 days post-injection (Fig. 6c). Moreover, the body weights of T24 xenograft mice demonstrated no impact after intravenous administration of NLS-FF-T (Fig. S25), indicating the high *in vivo* biocompatibility of the IAS system due to the encapsulated ATP-binding site inside nanoparticles. Additionally, the survival curves exhibited a strong correlation with the tumor growth results and the median survival time (Med sur.) of NLS-FF-T treated T24 xenograft mice was significantly prolonged compared to that of PBS (Med sur. = 55 days), NLS-FF (Med sur. = 61 days), FF-T (Med sur. = 60.5 days) and NLS-T (Med sur. = 65.5 days) treated mice, respectively (Fig. 6d). In summary, the excellent antitumor efficacy on residual tumor recurrence and low systemic toxicity of the IAS system highlights its potential in clinical translational application by induction of mitochondriopathy-like damages.

### *In vivo* antitumor efficacy of the IAS system in T24 orthotopic xenograft model

To validate the therapeutic benefits of the IAS system, a T24 orthotopic bladder cancer mouse model was developed to simulate the human condition [36]. The timeline of construction and treatment of T24 orthotopic bladder cancer mice are illustrated in Fig. 6e. In brief, the mice were treated intravenously with PBS, NLS-FF, FF-T, NLS-T and NLS-FF-T (500 μM in 200 μL PBS) at 2-day intervals for a total of six times (Fig. 6e). Subsequently, the tumor growth in each group was monitored using time-dependent bioluminescence signals of orthotopic bladder cancer mice observed at 0, 5, 10, 15 and 20 days post-injection. No significant differences in bioluminescence intensity were detected among the PBS, NLS-FF, FF-T, NLS-T and NLS-FF-T groups at 0 days post-injection (Fig. 6f), which suggested that the orthotopic bladder cancer mice model was established successfully. However, a rapidly amplified bioluminescence signal was observed in the PBS and NLS-FF treatment group after 5 days. Meanwhile, a moderate increase in the bioluminescence signal was detected in FF-T and NLS-T treatment groups from days 5 to 20. On the other hand, the bioluminescence intensity in NLS-FF-T treated mice steadily decreased and was almost unidentifiable at the end of treatment (Fig. 6g), exhibiting the potential of ATP sequestration-based NLS-FF-T in the efficient control of tumor growth. Furthermore, the survival curves exhibited a strong correlation with the tumor growth results, as shown in Fig. 6h. The survival time of NLS-FF-T treated mice was significantly extended compared to that of PBS, NLS-FF, FF-T and NLS-T treated mice. Therefore, these results demonstrate the outstanding antitumor effect of the IAS system through induction of mitochondriopathy-like damages in bladder cancer therapy.

### Biosafety and toxicity profile evaluation of the IAS system

To evaluate the potential *in vivo* side-effects of the IAS system, healthy BALB/c nude mice (approximately 6–8 weeks old, 16–18 g) were subjected to intravenous treatment with PBS or NLS-FF-T (1000 μM, 500 μM or 250 μM in 200 μL PBS) (*N* = 4) six times at 2-day intervals. At 12 days after final injection, the major organs (liver and kidney) and blood of healthy BALB/c nude mice were collected for histological, blood chemistry and routine blood analyses. The hematoxylin and eosin (H&E) staining results in Fig. S26 reveal no signs of histological deterioration or damage in the main organs of both PBS and NLS-FF-T treated mice. Furthermore, blood biochemistry analysis in Fig. S27 showed that all the essential indicators, including alanine aminotransferase (ALT), aspartate aminotransferase (AST), alkaline phosphatase (ALP), blood urea nitrogen (BUN) and creatinine (CRE) levels, exhibited typical ranges in both groups. Additionally, the routine blood examination results, as shown in Fig. S28, showed no differences in red blood cell (RBC), white blood cell (WBC), platelets (PLT), hemoglobin (HGB) and neutrophil (NEUT) levels between the PBS and NLS-FF-T treated groups. These results demonstrate that the IAS system has remarkable *in vivo* biocompatibility, while extending the therapeutic dose window.

## CONCLUSION

Leveraging the benefits of *in vivo* self-assembled nanomaterials, we designed an IAS system to construct nanofibrous nanostructures on the surface of tumor nuclei with exposed ATP binding sites, which leads to the efficient suppression of bladder cancer by inducing mitochondriopathy-like damages. The NLS-FF-T (HCA-K(T)LVFF-PEG-VKRKKKP) peptide was synthesized, comprising five distinct functional motifs, including coumarin (HCA), thymine (as the ATP-selective sequestering moiety), KLVFF (a β-sheet-forming peptide domain derived from β-amyloid peptide), PEG (as a hydrophilic surface to induce the formation of nanoparticles), and VKRKKKP (as the KPNA2-binding domain derived from the primary sequence of NLS). Three controls were also synthesized, including NLS-FF (HCA-KLVFF-PEG-VKRKKKP), FF-T (HCA-K(T)LVFF-PEG2) and NLS-T (HCA-K(T)LV-PEG-VKRKKKP). The reported NLS-FF-T self-assembled into nuclear-targeted nanoparticles with an ATP-binding site encapsulated inside under aqueous conditions. Upon interaction with KPNA2, the NLS-FF-T transformed into nanofibrous-based ATP traps on the surface of tumor nuclei, hindering intracellular energy production. The IC50 of NLS-FF-T was found to be ∼4-fold lower than that of NLS-T in multiple bladder tumor cell lines (T24, EJ and RT-112). Following intravenous administration, NLS-FF-T was dose-dependently accumulated at the tumor site of T24 xenograft mice and exhibited an excellent antitumor efficacy, leading to the deterioration of T24 tumors and prolonged overall survival of T24 orthotopic xenograft mice. These findings demonstrated the therapeutic benefits of induction of mitochondriopathy-like damages by intracellular ATP sequestration in treating malignancies.

## METHODS

### Synthesis and characterization of the IAS system

Experiments used peptide (HCA-K(T)LVFF-PEG-VKRKKKP) synthesized by standard Fmoc solid-phase peptide synthesis. Briefly, the NLS-FF-T was prepared by standard Fmoc solid-phase peptide synthesis, followed by conjugation with HCA or thymine. Briefly, the Pro resin (loading: 0.39 mM/g) was used for the phase supports, then repeated deprotection (piperidine: 20% v/v) and acrylation (*N*-methylmorpholine (NMM): 4%, 2-(1*H*-benzotriazol-1-yl)-1,1,3,3-tetramethyluronium hexafluorophosphate (HBTU): 6%) in anhydrous *N,N*-dimethylformamide (DMF). After deprotection of the Fmoc of the last amino acid, HCA or thymine was dissolved in DMF (NMM: 4%) and further reacted with the peptide. The NLS-FF-T is cleaved from resin with the mixture of trifluoroacetic acid (TFA), Triisopropylsilane (TIPS) and H_2_O in a volume ratio of 95 : 2.5 : 2.5 for 2.5 hours. The final solution was concentrated, precipitated in ether, and purified by reverse-phase high-performance liquid chromatography (HPLC). The molecular weight and purity of NLS-FF-T were characterized by matrix-assisted laser desorption/ionization time-of-flight mass spectrometry (MALDI-TOF-MS) and HPLC. NLS-FF (HCA-KLVFF-PEG-VKRKKKP), FF-T (HCA-K(T)LVFF-PEG2) and NLS-T (HCA-K(T)LV-PEG-VKRKKKP) were prepared by similar procedures.

### Molecular dynamics simulations

In this work, two different all-atom molecular dynamics simulations were performed to reveal the interaction between self-assembled β-sheets and KPNA2 (PDB ID: 4WV6) [37], and the absorption of ATP molecules (PDB ID: 7RGQ) [38] on the β-sheet-Protein Complex. The initial structures of residue X and Y of the β-sheet were initially generated by Gaussian View, and then optimized in Gaussian with b3lyp [39]/def2-tvzp method. The force field parameters of residue X/Y were fitted based on the General AMBER force field (GAFF) [40] by the antechamber of amber [41]. Also, the β-sheet monomer was generated by antechamber based on the predefined backbone. Subsequently, the assembled β-sheets were built in PyMol [42]. For KPNA2, the missed residues from the crystal structure were completed by Modeller [43], and the protonation states of the residues were decided with H++ [44]. Three KPNA2 proteins were docked to the assembled β-sheets, and the complex was solvated in a 0.15 M NaCl solution, including 125 699 TIP3P waters [45], 364 Na^+^ and 385 Cl^−^ [46]. The β-sheet-KPNA2 system was temperature coupled with a V-rescale thermostat to a bath of 298.15 K in a 1 ns NVT equilibrium simulation and then pressure coupled with a semi-isotropic Berendsen barostat to 1 bar in a 2 ns NPT simulation [47]. Then, 120 ns production run was performed for the β-sheet-KPNA2 system with frame recorded every 2 ps.

Similarly, the ATP molecule was optimized in Gaussian with b3lyp[39]/def2-tvzp method, and the force field parameters were generated by antechamber [40,41]. Then, 12 ATP molecules were docked to the terminal of residue X for each β-sheet by following the best H-bond network formation. After that, the β-sheet-KPNA2-ATP complex was solvated in a 0.15 M NaCl solution that contains 186 953 TIP3P water molecules [45], 534 Na^+^ and 555 Cl^−^ [46]. Afterward, the equilibrium simulations for the system were finished, including 1 ns NVT and 2 ns NPT calculation. Finally, the production run of the simulation was performed for ∼20 ns and the frame recorded with the same frequency. All the molecular dynamic simulations were performed by GROMACS-2019.4 [48], and all the structures are displayed by VMD [49].

## Supplementary Material

nwae028_Supplemental_File
