# Supplementary material for: Inducing mitochondriopathy-like damages by transformable nucleopeptide nanoparticles for targeted therapy of bladder cancer
Source: Natl Sci Rev. 2024 Jan 22;11(4):nwae028. doi: 10.1093/nsr/nwae028 (PMC10903983; doi:10.1093/nsr/nwae028)
Supplement: nwae028_Supplemental_File [file nwae028_supplemental_file.pdf]

RESEARCH ARTICLE

MATERIALS SCIENCE

Inducing mitochondriopathy-like damages by transformable nucleopeptide nanoparticles for targeted therapy of bladder cancer

Da-Yong Hou[#,a,b], Ni-Yuan Zhang[#,c], Lu Wang[#,a,b], Mei-Yu Lv[a], Xiang-Peng Li[a,b], Peng Zhang[a,b], Yue-Ze Wang[a,b], Lei Shen[d], Xiu-Hai Wu[a,b], Bo Fu[a,b], Peng-Yu Guo[a,b], Zi-Qi Wang[a,b], Dong-Bing Cheng[*,d], Hao Wang[*,c] and Wanhai Xu[*,a,b]

[a]NHC and CAMS Key Laboratory of Molecular Probe and Targeted Theranostics, Heilongjiang Key Laboratory of Scientific Research in Urology, Harbin Medical University, Harbin 150001, China;

[b]Department of Urology, Harbin Medical University Cancer Hospital, Harbin 150001, China;

[c]CAS Key Laboratory for Biomedical Effects of Nanomaterials and Nanosafety, CAS Center for Excellence in Nanoscience, National Center for Nanoscience and Technology (NCNST), Beijing 100190, China;

[d]School of Chemistry, Chemical Engineering & Life Science, Hubei Key Laboratory of Nanomedicine for Neurodegenerative Diseases, Wuhan University of Technology, Wuhan 430070, China

*__Corresponding authors.__ E-mails: xuwanhai@hrbmu.edu.cn; wanghao@nanoctr.cn; chengdb@whut.edu.cn

#Equally contribute to this work.

Supplementary Methods

Materials

2-(4-Amidinophenyl)-6-indolecarbamidine dihydrochloride (DAPI, D8200) were purchased from Beijing Solarbio Science & Technology Co., Ltd. (Beijing, China). Penicillin and streptomycin (CK0008-100ML) CCK-8 Cell Proliferation and Cytotoxicity Assay Kit (CK0001-100T) and 4% paraformaldehyde (CK0014-500ML) were purchased from Beijing Chengzhi Kewei Biotechnology Co. Ltd. D-Luciferin potassium salt (D12505) and Hoechst 33342 (B2662) were purchased from LABLEAD Inc. (Beijing, China). Bax (D2E11) Rabbit mAb, Bcl-2 (D55G8) Rabbit mAb and Caspase-3 (D3R6Y) Rabbit mAb and β-Actin (8H10D10) Mouse mAb were purchased from Cell Signaling Technology (CST, USA). T24, EJ and RT-112 cell lines were purchased from the Cell Culture Center of the Institute of Basic Medicine, Chinese Academy of Medical Sciences (Beijing, China). Female BALB/c nude mice (8 weeks old, 18-20 g) were purchased from Vital River Laboratory Animal Technology Co., Ltd. (Beijing, China).

Characterization of IAS system

The chemical structures of peptides in **IAS** system were analyzed by MALDI-TOF-MS and ^{1}H NMR spectra. MALDI-TOF-MS were performed on a mass spectrometer (Bruker Daltonics, Germany) and recorded as an average of 500 laser shots. ^{1}H NMR spectra were obtained from a Bruker ARX 400 MHz spectrometer using DMSO-d6 as the solvent. Transmission electron microscopy (TEM, Tecnai G2 20 S-TWIN) was conducted to observe the morphology and diameters of molecules in IAS system in aqueous solution, and the particle sizes of nanoparticles are monitored by DLS (Nano-ZS 3600). Circular dichroism (CD) spectra are obtained by using a J 1500 CD spectrometer (Jasco, Japan). Fourier-transform infrared (FTIR) spectra were recorded on a PerkinElmer-2 spectrometer using KBr pellets. F-280 spectrofluorometer is applied for fluorescence analysis.

Morphology transformation responding to karyopherin KPNA2 of IAS system

To investigate morphology transformation of **NLS-FF-T** responding to KPNA2, the **NLS-FF-T** (10 μM) was dissolved in PBS with KPNA2 (0.5 μg mL^{-1}) and gently stirred at 37°C. The morphology transformation of **NLS-FF-T** assemblies are analyzed by TEM and DLS at certain time intervals. The β-sheet structure of **NLS-FF-T** nanofibers were confirmed by CD spectra and FTIR.

Determination of critical aggregation concentration (CAC)

To determine the Critical Aggregation Concentration (CAC) of **NLS-FF-T**, pyrene was utilized as a fluorescent probe. Initially, 50 μL of pyrene solution in acetone (480 μM) was added to a 5 mL centrifuge tube and the acetone was allowed to evaporate completely. A range of **NLS-FF-T** solutions with different concentrations were added to the tube while the concentration of pyrene was kept constant at 6 μM. The excitation spectra were recorded at an emission wavelength of 393 nm and the intensity ratio (I338/I335) was analyzed as a function of **NLS-FF-T** concentration. The CAC was determined by identifying the intersection point at low concentration on the plot.

Animals and cells

All animal procedures were carried out in compliance with ethical regulations and were approved by the Institutional Animal Care and Use Committee of the National Center for Nanoscience and Technology (NCNST21-2208-0609). Female BALB/c nude mice (about 6-8 weeks old) were procured from Vital River Laboratory Animal Technology Co., Ltd. (Beijing, China) and housed in ventilated cages (N=5) with a 12-h light-dark cycle (8:00-20:00 light/20:00-8:00 dark), constant room temperature (22 ℃) and relative humidity (30-70%), with ad libitum access to food and water. The T24 cell lines were obtained from the Cell Culture Center of the Institute of Basic Medicine, Chinese Academy of Medical Sciences (Beijing, China) and were cultured in Dulbecco's Modified Eagle Medium (DMEM) with high glucose containing 10% fetal bovine serum and 1% penicillin sulfate and streptomycin at 37 ℃ with 5% CO_2.

*Circular dichroism (CD) spectra of **IAS** system*

The secondary structure of the **NLS-FF-T** was monitored by a circular dichroism (CD) spectrum (JASCO Corporation, JC-1500). **NLS-FF-T** (0.5 mg/mL) was dissolved in PB solutions and kept at 37 ℃ under constant shaking. The CD signals were recorded at 24 h. CD Pro was utilized to analyze the β-sheet percentage in the secondary structure of **NLS-FF-T**. The percentage results of **NLS-FF-T** with or without KPNA2 were calculated by the three standard algorithms (CONTINLL, SELCON3, CDSSTR).

*Fourier transform infrared (FTIR) spectroscopy measurement of **IAS** system*

Molecular arrangement of **NLS-FF-T** was analyzed by FTIR. The solution of **NLS-FF-T** (0.5 mg/mL) was incubated with or without KPNA2 for 8 h, and dialyze (MWCO: 3500 Da) against PB for 48 h. The resulting solution was lyophilized for FTIR characterization.

Evaluation ATP sequestration ability of ***IAS*** *system*

To evaluate the ATP sequestration ability of **IAS** system, ATP hydrolysis rate in alkaline phosphatase (ALP) following treatment with **NLS-FF-T** was conducted. Initially, ATP (200 μM) was mixed with **NLS-FF-T** nanofibers or nanoparticles and dissolved in DEA buffer (10 mM, pH 9.8). The mixture was then incubated for varying amounts of time followed by inactivation at 75 ℃ for 5 min. The remaining ATP was determined using an ATP assay kit, which utilized the generated chemiluminescence to calculate ATP hydrolysis rate. All hydrolysis reactions were conducted at 37 ℃ with continuous agitation.

Cytotoxicity assay of ***IAS*** *system*

The Cell Counting Kit-8 (CCK-8) assay was utilized to evaluate the cytotoxicity of NLS-FF, FF-T, NLS-T and **NLS-FF-T** against T24 cells. Initially, 100 μL of T24 cell suspension was seeded into a 96-well plate at a density of 5×10^3 cells/mL and cultured overnight in a humidified environment containing 5% CO_2 at 37 ℃. Subsequently, DMEM culture medium containing NLS-FF, FF-T, NLS-T and **NLS-FF-T** at a predetermined range of concentrations (3.125, 6.25, 12.5, 25, 50, 100, 200, 400, 800 and 1600 μM) was incubated with T24 cells at 37 ℃ for 24 h. Next, the T24 cells in the 96-well plate were washed with a PBS solution and then treated with 100 μL of freshly prepared 10% CCK-8 solution in cell culture medium. The absorbance of CCK-8 was measured using a microplate reader.

Cellular imaging study of ***IAS*** *system*

To begin, a 1 mL cell suspension of T24 cells was seeded in a confocal imaging chamber at a density of 5×10^5 cells/mL and cultured for one night in a humidified environment containing 5% CO_2 at 37 ℃. Following this, the T24 cells were incubated with NLS-FF, FF-T, NLS-T and **NLS-FF-T** at a concentration of 50 μM in serum-free cell culture medium at 37 ℃ for 2 h. Next, the T24 cells were washed with PBS buffer three times and fixed with 4% paraformaldehyde for 30 min. Finally, the T24 cells were imaged using a Confocal Laser Scanning Microscope (CLSM) under 40 × objective (Ex/Em: 640 nm/725 nm).

Protein extraction and western blotting

The T24 cells were incubated with PBS, NLS-FF, FF-T, NLS-T and **NLS-FF-T** at a concentration of 50 μM at 37°C. Afterward, the cells were homogenized in RIPA extraction buffer (LABLEAD Inc., China, R1091) and centrifuged at 14,000 g for 15 minutes at 4 ℃. The protein concentrations in the supernatant were measured using the BCA Protein Assay Kit according to the manufacturer's instructions. The samples were then separated by SDS polyacrylamide gel electrophoresis (SDS-PAGE) and transferred onto polyvinylidene difluoride (PVDF) membranes. The PVDF membranes were blocked with NcmBlot blocking buffer (NCM Biotech) for 10 minutes, followed by incubation with primary antibodies and corresponding secondary antibodies overnight. Finally, detection and imaging were performed using a chemiluminescence imaging system with HRP substrate.

Intracellular ATP levels assay

A 2 mL suspension of T24 cells was seeded at a density of 5×10^5 cells per well in a 6 well-plate and cultured overnight in a humidified environment containing 5% CO_2 at 37 ℃. The T24 cells were then incubated with NLS-FF, FF-T, NLS-T and **NLS-FF-T** at a concentration of 50 μM in serum-free cell culture medium at 37 ℃ for 12 h. After lysing by homogenization, the T24 cell samples were collected by centrifugation at 10000 g for 10 min at 4 ℃. Finally, the ATP levels were measured using ATP Content Assay Kit in accordance with the manufacturer's instructions.

Intracellular morphology observation of ***IAS*** *system*

T24 cells were collected by centrifugation at 3000 rpm for 5 min after administration of **NLS-FF-T** (50 μM). The collected cells were fixed with 2.5% glutaraldehyde at 4°C overnight and then treated with 1% osmium dichromate for 2 h after three rinses with PBS. Dehydration process was carried out with ascending concentrations of alcohol solutions (50, 70, 80, 90, 100, 100, and 100%) for 10 min each. The cells were then exposed to a mixture of alcohol/acetone (1:1) for 10 min, subsequently, a mixture of acetone/EPON-812 (1:1) and then a mixture of acetone/EPON-812 (1:2) for 1 h each. Pure EPON-812 was used for further cell sample infiltration overnight at 4 ℃. The curative binding of EPON-812 with cells was carried out at 37, 45, and 60 ℃ for 24 h respectively. Finally, the intracellular morphology of **NLS-FF-T** was observed by Transmission Electron Microscopy (TEM) after staining with 2% uranyl acetate and 3% lead citrate.

Immunohistochemistry (IHC) assay

To investigate the expression of KPNA2 in bladder tumor tissues from 30 patients combined with normal bladder tissues from 20 patients, immunohistochemistry (IHC) was conducted by Wuhan Servicebio Technology Co., Ltd. Fresh bladder cancer specimens and normal bladder urothelium specimens were collected and fixed for analysis after appropriate informed consent was obtained. The experiments performed using human specimens were reviewed and approved by the Committees for Ethical Review of the Fourth Hospital of Harbin Medical University (2022-SCILLSC-27).

Microscale Thermophoresis (MST) ligand binding measurements.

To investigate the binding affinity of FF-T and **NLS-FF-T** with KPNA2, Protein Labeling Kit RED-NHS was initially used to label KPNA2 following the manufacturer's instructions. The labeled KPNA2 was then incubated with concentration gradients of FF-T and **NLS-FF-T**. Finally, the prepared samples were loaded into silica capillaries and analyzed with Monolish NT.115.

In vivo dose-dependent fluorescence imaging of ***IAS*** *system*

To construct T24 xenograft mice, a density of 5×10^6 T24 cells resuspended in Matrigel (Corning, 354248) were subcutaneously injected into the right hind of BALB/c nude mice (approximately 6-8 weeks, 16-18 g). When the average tumor volume reached around 200-400 mm^3, the T24 xenograft mice were intravenously injected with **NLS-FF-T** (500 μM or 250 μM in 200 μL PBS) for *in vivo* fluorescence imaging at different time points (1, 4, 8, 12, 24, 36, 48, 60, 72, 84 and 96 h) with an *in vivo* imaging system (IVIS) Spectrum.

Ex vivo dose-dependent fluorescence imaging of ***IAS*** *system*

To construct T24 xenograft mice, a density of 5×10^6 T24 cells resuspended in Matrigel (Corning, 354248) were subcutaneously injected into the right hind of BALB/c nude mice (approximately 6-8 weeks, 16-18 g). When the average tumor volume reached around 200-400 mm^3, Cy labeled **NLS-FF-T** was administered to the T24 xenograft mice at various concentrations (500 μM, 250 μM, 125 μM or 62.5 μM in 200 μL PBS) via intravenous injection. Finally, the T24 xenograft mice were sacrificed, of which the main organs and tumors were collected for *ex vivo* fluorescence imaging of **NLS-FF-T**.

Intratumoral morphology observation of ***IAS*** *system*

After intravenous administration of **NLS-FF-T** (500 μM in 200 μL PBS) in T24 xenograft mice, tumor tissues were collected and fragmented into small pieces measuring 3 ×

3 mm at 8 h post-treatment. The fragments were then fixed with 2.5% glutaraldehyde at 4 ℃ overnight and treated with 1% osmium dichromate for 2 h after being rinsed thrice with PBS. Subsequently, graded concentrations of alcohol solutions (50, 70, 80, 90, 100, 100 and 100%) were utilized for graded dehydration (with 10 minutes each). Next, tumor fragments were exposed to a mixture of alcohol/acetone (1:1) for 10 min, a mixture of acetone/EPON-812 (1:1) for 1 h and a mixture of acetone/EPON-812 (1:2) for 1 h. Pure EPON-812 was then utilized to further infiltrate the tumor fragments overnight at 4 ℃, after which it was cured at 37, 45 and 60 ℃ for 24 h respectively. Finally, the morphology of **NLS-FF-T** in the tumor slice was observed by bio-Transmission Electron Microscope (bio-TEM) after being stained with 2% uranyl acetate and 3% lead citrate.

In vivo antitumor evaluation of ***IAS*** *system in subcutaneous xenograft model*

To further verify the therapeutic potency of the **IAS** system, a T24 xenograft mouse model was established by subcutaneously injecting T24 cells (5×10^6) suspended in 100 μL Matrigel into the right flank of BALB/c nude mice (approximately 6-8 weeks, 16-18 g). When the average tumor volume reached 100-200 mm^3, the T24 tumor-bearing mice were randomly divided into five groups (with N=6 mice per group) for treatments with PBS, NLS-FF, FF-T, NLS-T and **NLS-FF-T** (500 μM in 200 μL PBS) six times via intravenous injection on days 0, 2, 4, 6, 8 and 10, respectively. The individual tumor growth and body weight were measured every 2 d for analysis, with tumor volume calculated as follows: tumor width^2 × tumor length × 0.5. Additionally, all tumor tissues were harvested intact and weighed at the end of the treatment period.

Tumor ATP levels assay

At the end of the treatment period, T24 tumor tissues were collected and washed thrice with PBS. Subsequently, the tumor lysates were gathered by homogenization and centrifuged at 8000 g for 10 min at 4 ℃. The ATP levels were then measured according to the manufacturer's instructions using an ATP Content Assay Kit.

Immunofluorescence and immunohistochemistry assay

At the end of the treatment period, T24 tumor tissues were collected and fixed with 4% paraformaldehyde for analysis. Subsequently, Wuhan Servicebio Technology Co., Ltd. performed hematoxylin-eosin (H&E) staining, terminal deoxynucleotidyl transferase deoxyuridine triphosphate (dUTP) nick end labeling (TUNEL) assay and Ki67 immunohistochemical staining of sections of the T24 tumor tissue.

*In vivo antitumor evaluation of **IAS** system in regrowth of residual tumor model*

To establish the T24 xenograft mouse model, BALB/c nude mice (approximately 6-8 weeks, weighing 16-18 g) were subcutaneously injected with T24 cells (5×10^6) suspended in 100 μL Matrigel. The mice were randomly divided into five groups, with six mice in each group. Both body weight and tumor volume were measured for analysis. At 21 d after inoculation, tumor tissues of approximately 300-500 mm^3 were resected, leaving a residual tumor of approximately 30-50 mm^3. After that, the T24 xenograft mice were intravenously administered with PBS, NLS-FF, FF-T, NLS-T and **NLS-FF-T** (500 μM in 200 μL PBS) every two days on day 21, 23, 25, 27, 29 and 31. The tumor volume was calculated using the formula: tumor $width^2 \times$ tumor length $\times 0.5$.

*In vivo antitumor evaluation of **IAS** system in T24-Luc orthotopic bladder cancer mice*

In brief, BALB/c nude mice (approximately 6-8 weeks, 16-18 g) were anesthetized with isoflurane. The inner mucosa of bladder was slightly damaged using an IV catheter (24G), followed by the intravesical delivery of T24-Luc cells into the bladder cavity via the same catheter. After incubation for 60 min, the mice were subjected to bioluminescent imaging to confirm and monitor orthotopic tumor growth in the bladder. The mice were then randomly assigned to five groups (with N=6 mice per group) and intravenously administered with PBS, NLS-FF, FF-T, NLS-T and **NLS-FF-T** (500 μM in 200 μL PBS) every two days on day 0, 2, 4, 6, 8 and 10, respectively.

*In vivo toxicology evaluation of **IAS** system*

Healthy BALB/c nude mice (approximately 6-8 weeks, 16-18 g) were used to evaluate the *in vivo* toxicity of **IAS** system. **NLS-FF-T** was intravenously injected into four healthy mice each at doses of 1000 μM, 500 μM and 250 μM, six times on day 0, 2, 4, 6, 8 and 10, respectively. Four healthy mice treated with PBS were used as controls. On day 11, major organs including liver and kidney were excised for histological analysis by Hematoxylin and Eosin (H&E) staining, which was conducted by Servicebio Technology Co., Ltd. (Wuhan, China). Meanwhile, the mice were sacrificed for blood chemistry and blood routine analyses performed by Vital River Laboratory Animal Technology Co., Ltd. (Beijing, China).

Statistical methods

For two-group comparisons, statistical analysis was performed using one-way ANOVA followed by post hoc Tukey's test. The GraphPad Prism software 8.0 was used for all statistical analyses. Survival curves were generated using the Kaplan-Meier method and

compared using the log-rank test. All values presented in this study are expressed as mean ± standard deviation (SD). Statistical significance was indicated as NS for no significance, *$P < 0.05$, **$P < 0.01$, and ***$P < 0.001$.

Supplementary Figures

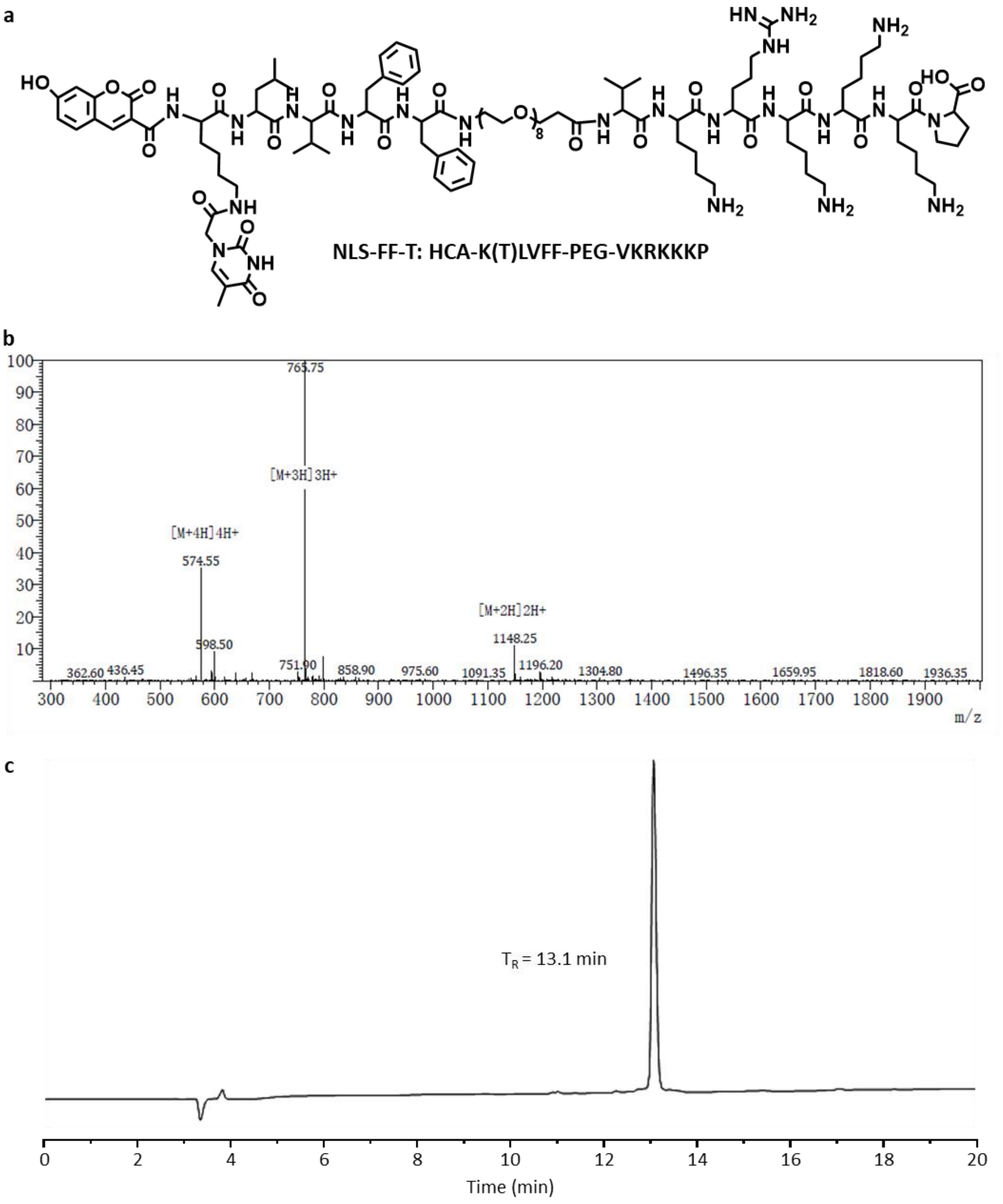

Figure S1. (a) The structure of **NLS-FF-T**; **(b)** MALDI-TOF of **NLS-FF-T**; **(c)** HPLC of **NLS-FF-T**.

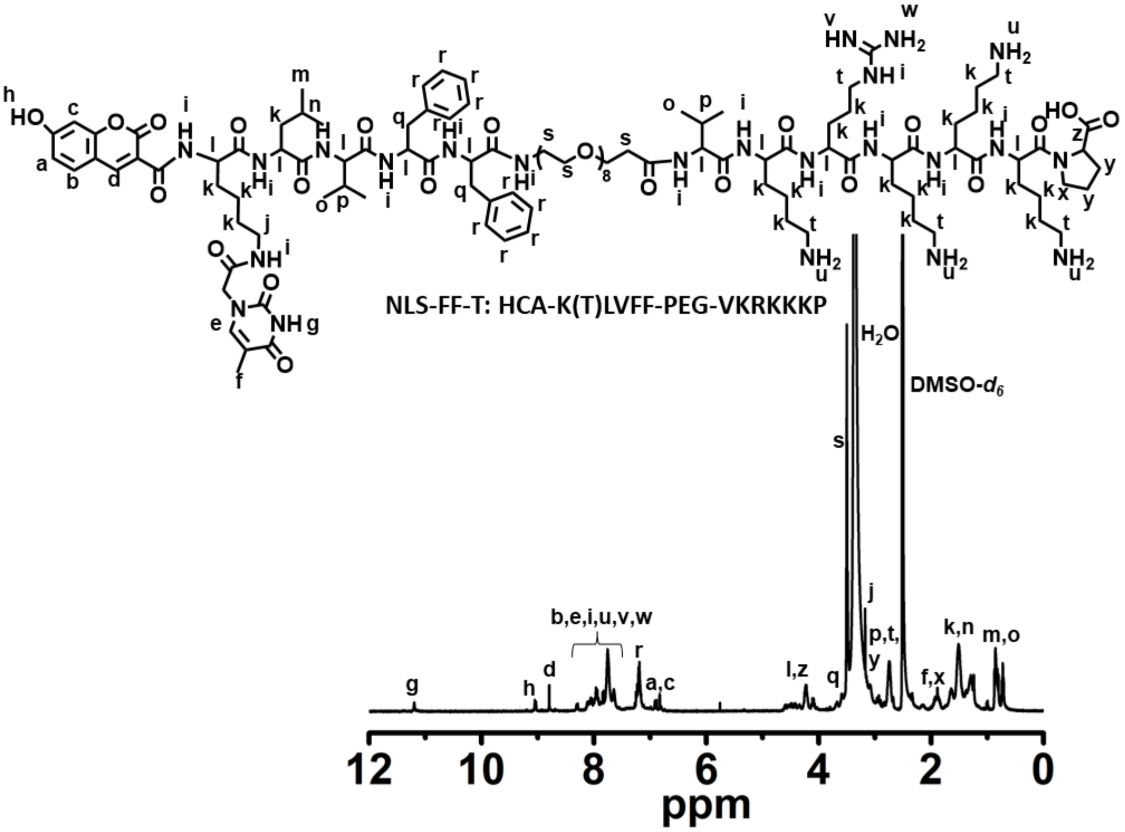

Figure S2. ^{1}H NMR (400 MHz, DMSO-d_6) spectrum of **NLS-FF-T**.

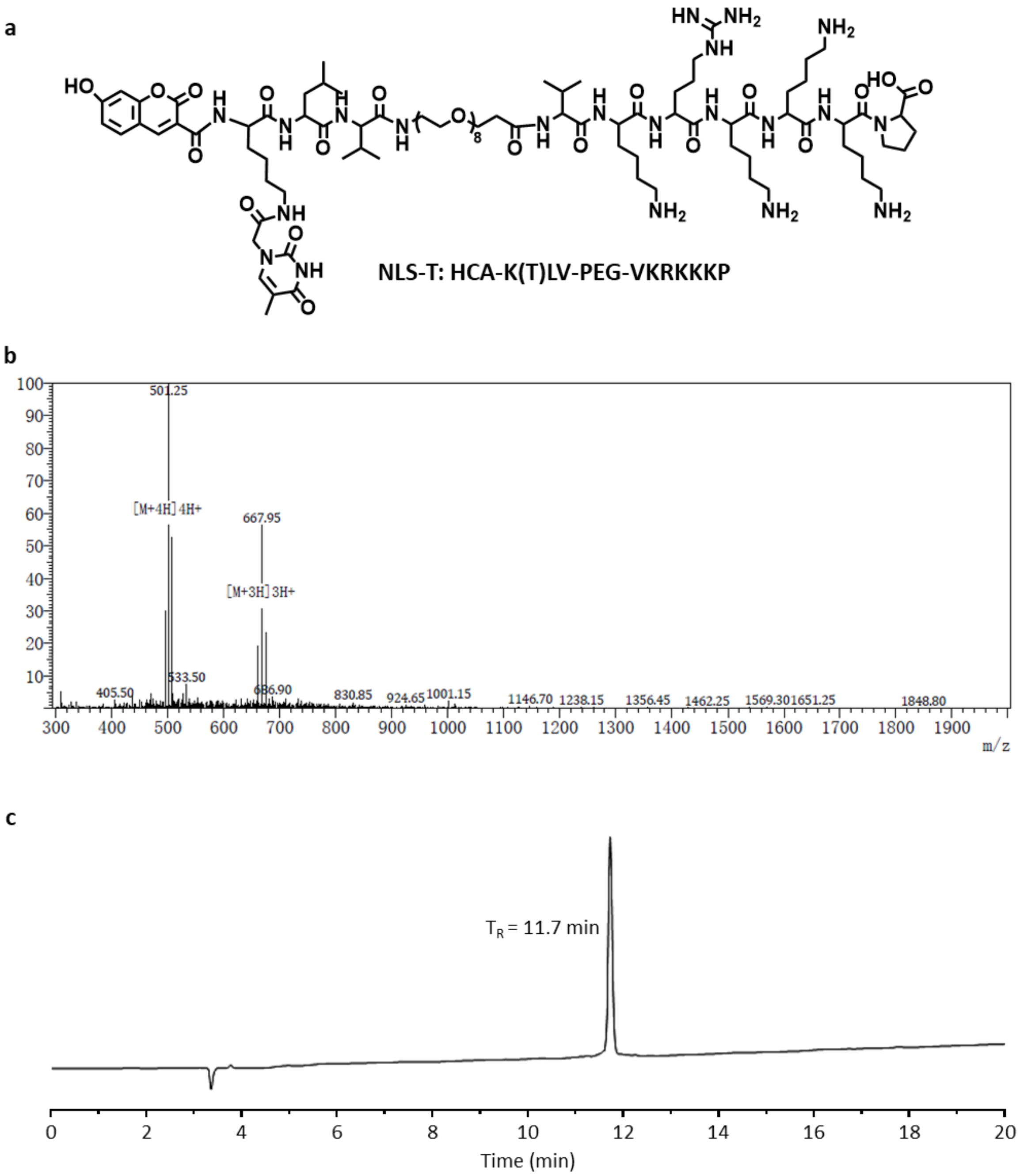

Figure S3. (a) The structure of **NLS-T**; **(b)** MALDI-TOF of **NLS-T**; **(c)** HPLC of **NLS-T**.

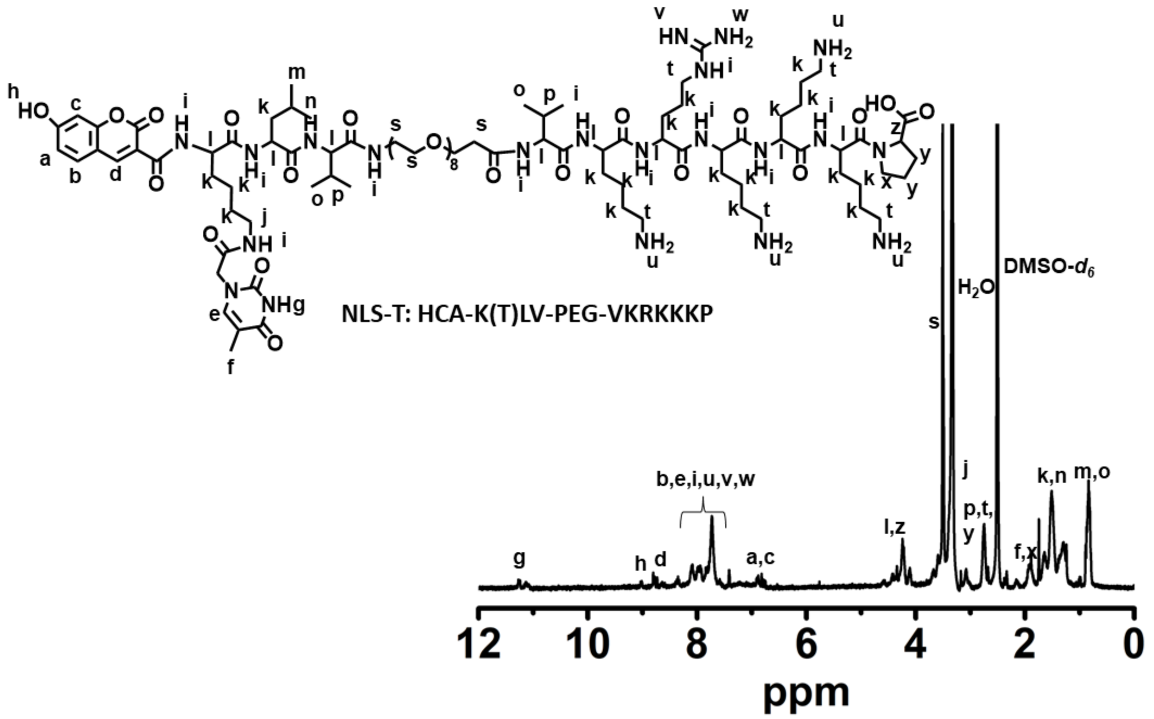

Figure S4. ^{1}H NMR (400 MHz, DMSO-d_6) spectrum of **NLS-T**.

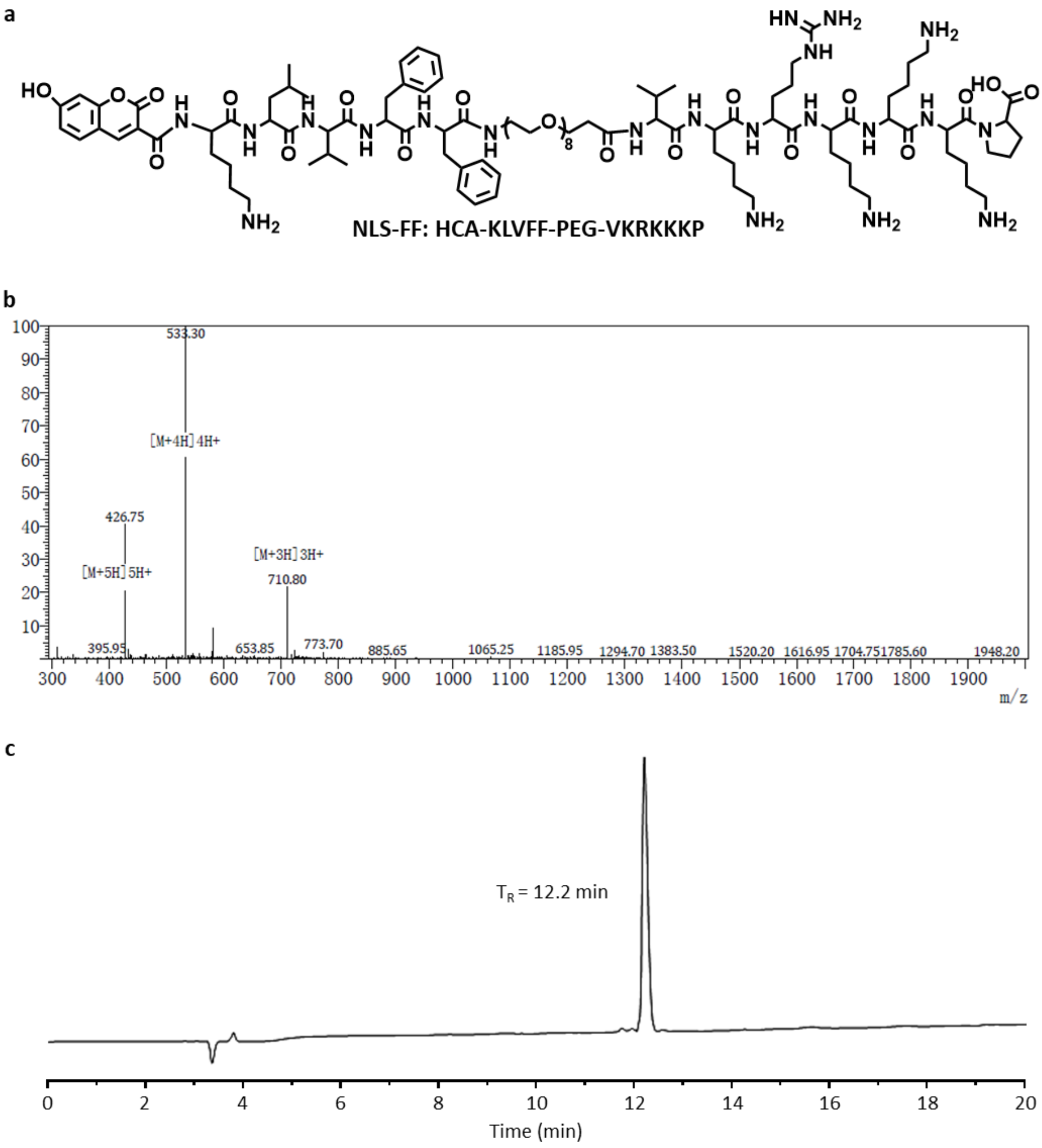

Fig. S5. (a) The structure of **NLS-FF**; **(b)** MALDI-TOF of **NLS-FF**; **(c)** HPLC of **NLS-FF**.

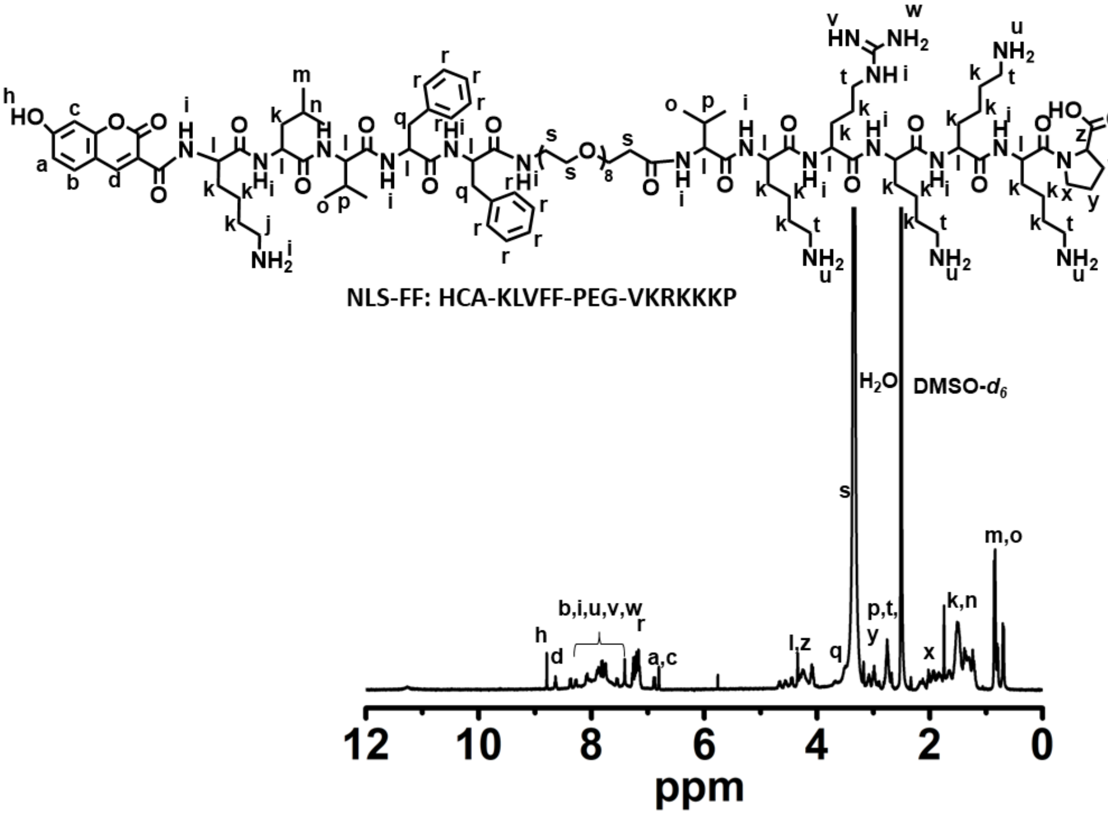

Fig. S6. ^{1}H NMR (400 MHz, DMSO-d_6) spectrum of **NLS-FF**.

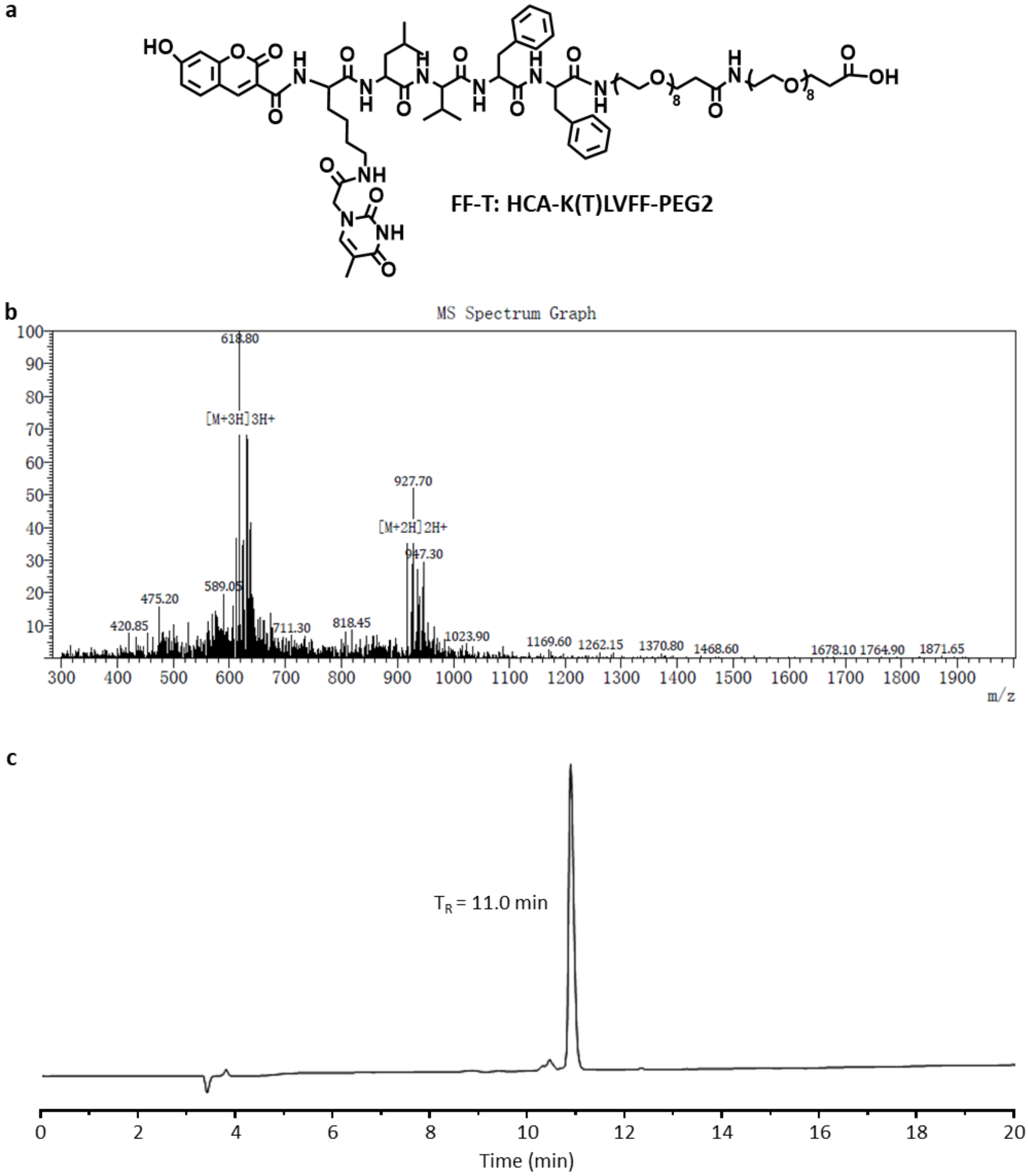

Fig. S7. (a) The structure of **FF-T**; **(b)** MALDI-TOF of **FF-T**; **(c)** HPLC of **FF-T**.

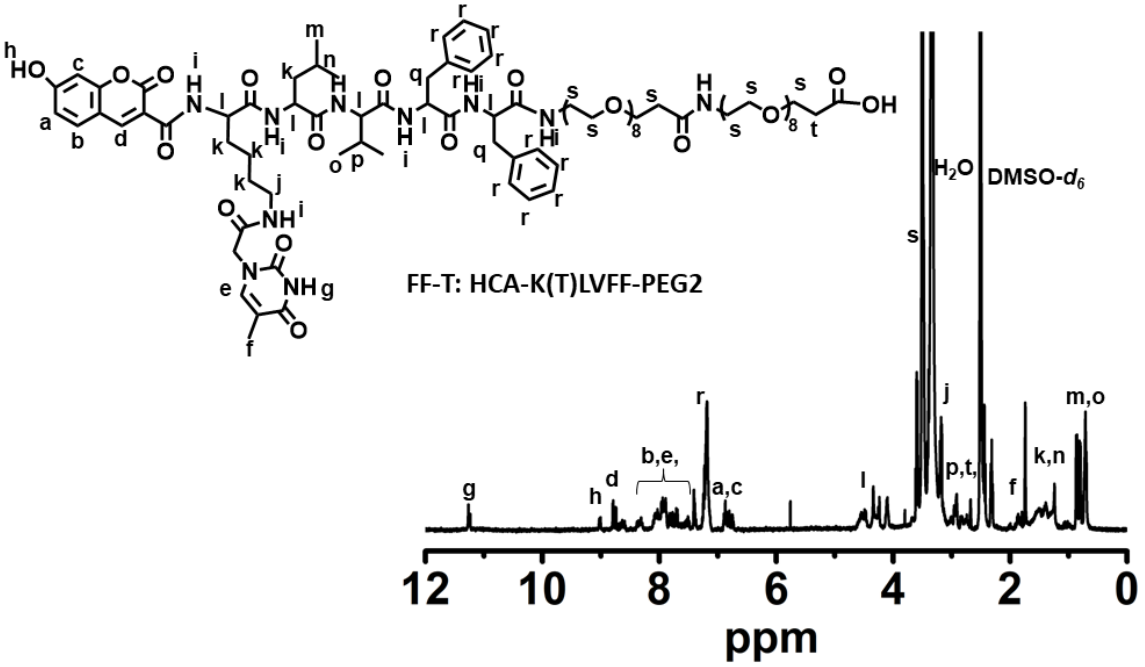

Fig. S8. ^{1}H NMR (400 MHz, DMSO-d_6) spectrum of **FF-T**.

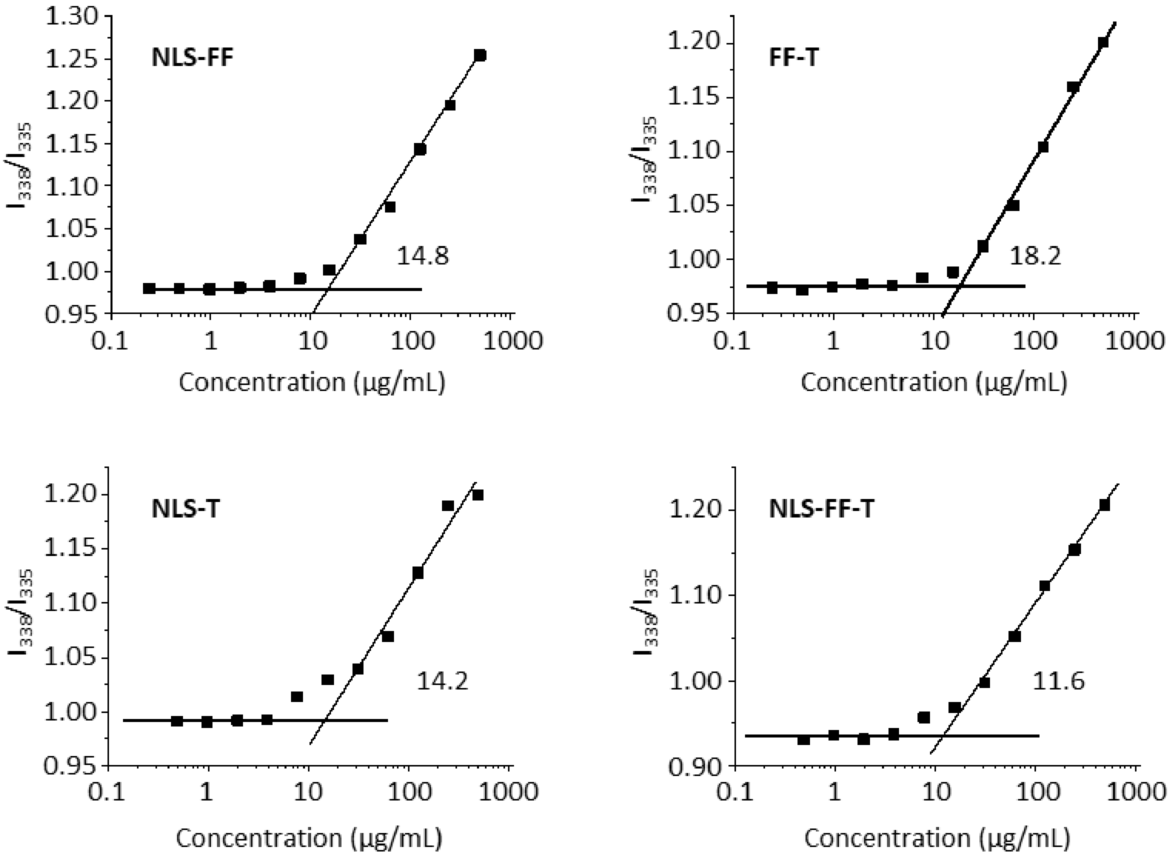

Fig. S9. The critical aggregation concentration (CAC) of NLS-FF, FF-T, NLS-T and **NLS-FF-T** was measured with pyrene.

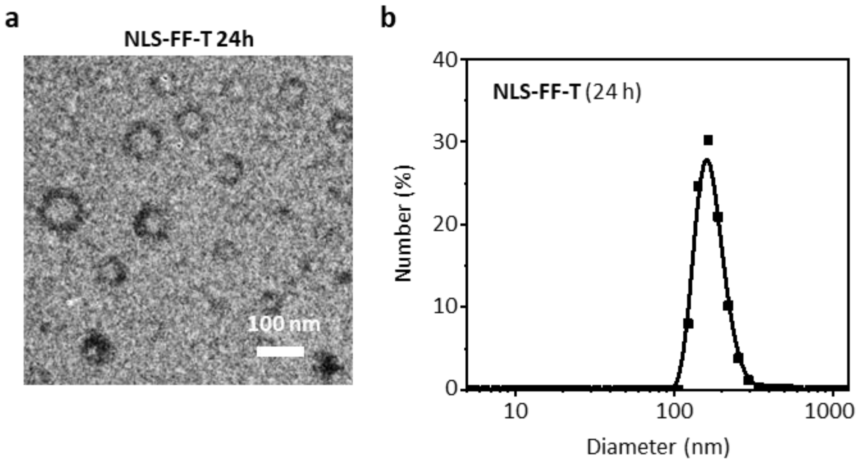

Fig. S10. (a) Representative TEM images of **NLS-FF-T** at 24 h. Scale bars: 100 nm. **(b)** Particle size of **NLS-FF-T** (24 h) measured by DLS.

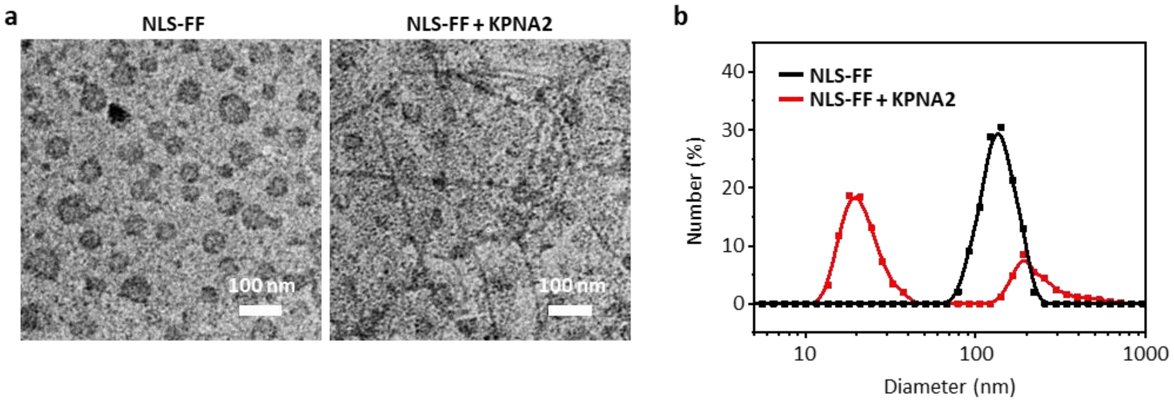

Fig. S11. (a) Representative TEM images of **NLS-FF** and **NLS-FF** transformed into nanofibrous after interaction with KPNA2 protein. Scale bars: 100 nm. **(b)** Particle size of **NLS-FF** and **NLS-FF** transformed into nanofibrous after interaction with KPNA2 protein measured by DLS.

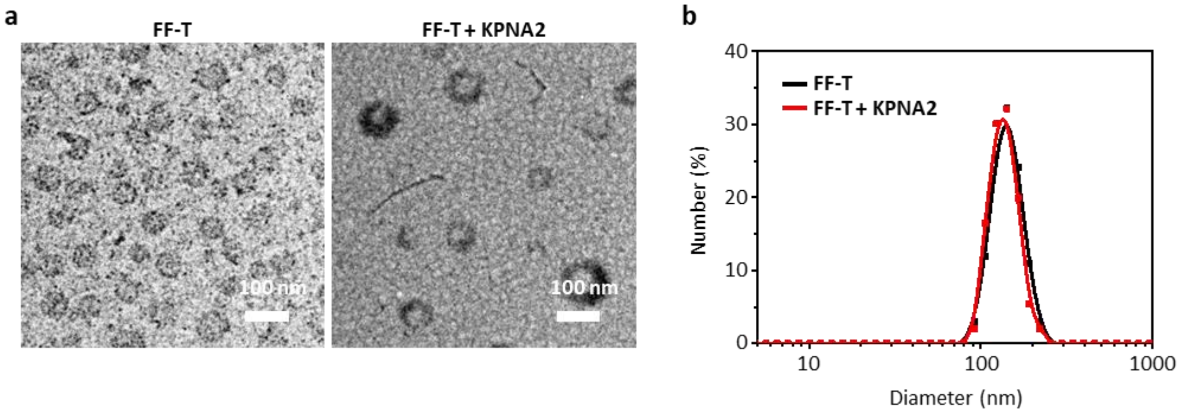

Fig. S12. (a) Representative TEM images of **FF-T** and **FF-T** interaction with KPNA2 protein. Scale bars: 100 nm. **(b)** Particle size of **FF-T** and **FF-T** interaction with KPNA2 protein measured by DLS.

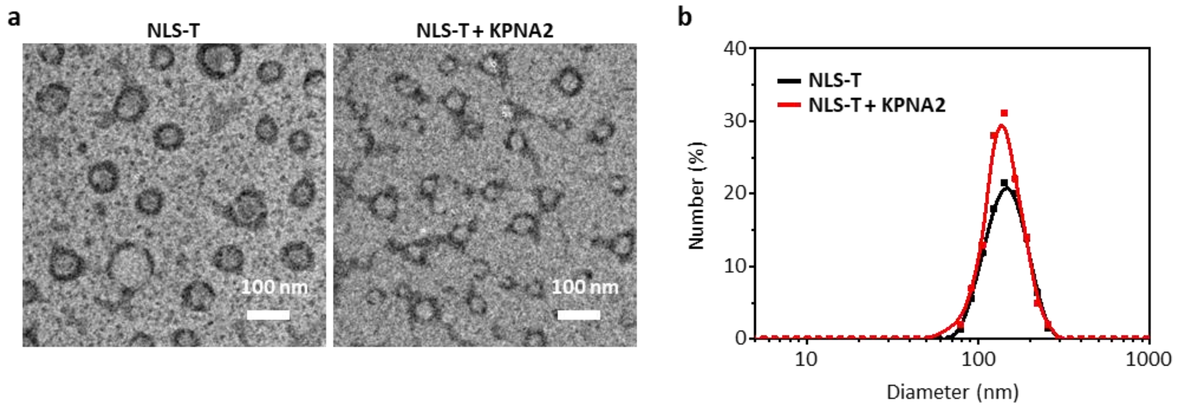

Fig. S13. (a) Representative TEM images of **NLS-T** and **NLS-T** interaction with KPNA2 protein. Scale bars: 100 nm. **(b)** Particle size of **NLS-T** and **NLS-T** interaction with KPNA2 protein measured by DLS.

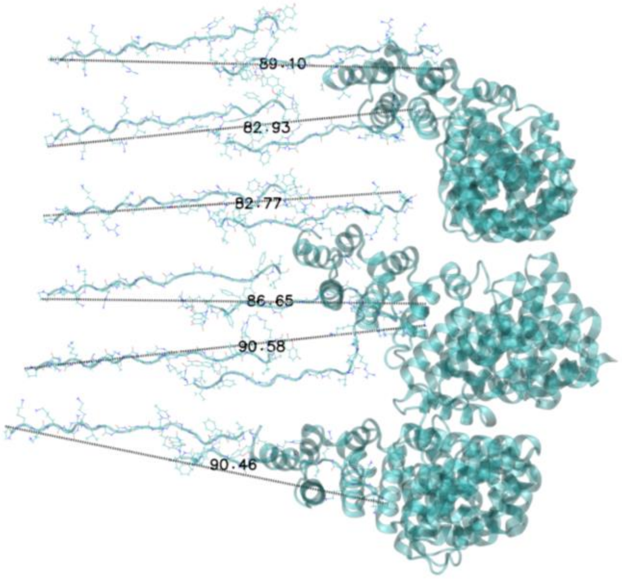

Fig. S14. The computational diameter of **NLS-FF-T** nanofiber.

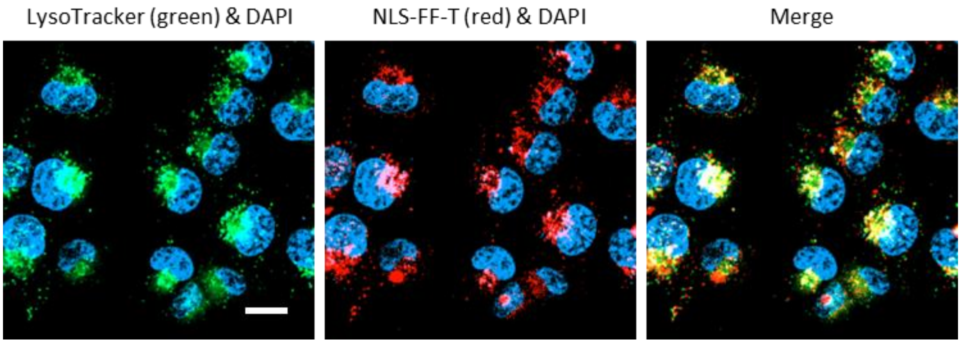

Fig. S15. Confocal laser scanning microscopy (CLSM) images of T24 cells after incubated with **NLS-FF-T** (red fluorescence signal). Lysosomes were labeled with LysoTracker Green. Scale bar: 10 μm.

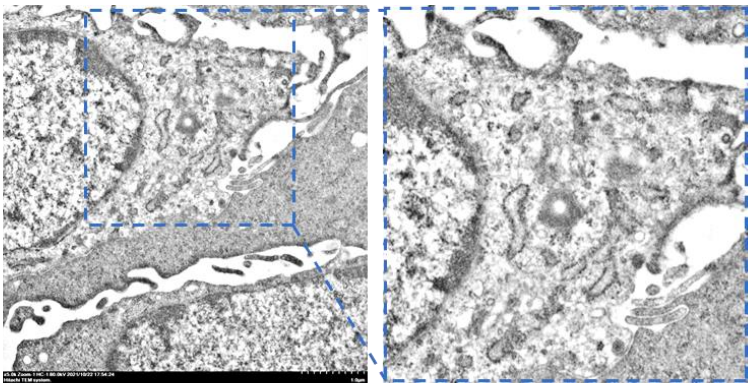

Fig. S16. Bio-TEM images of T24 cells after treated with PBS.

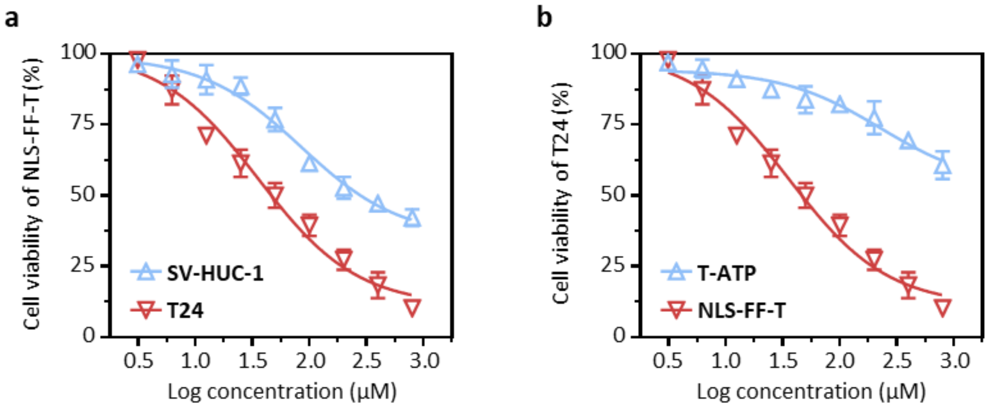

Fig. S17. (a) Viability of T24 and SV-HUC-1 cells after treated with **NLS-FF-T** at different concentrations for 48 h. **(b)** Viability of T24 cells after treated with **NLS-FF-T** or T-ATP (pure ATP sequestration motif) at different concentrations for 48 h.

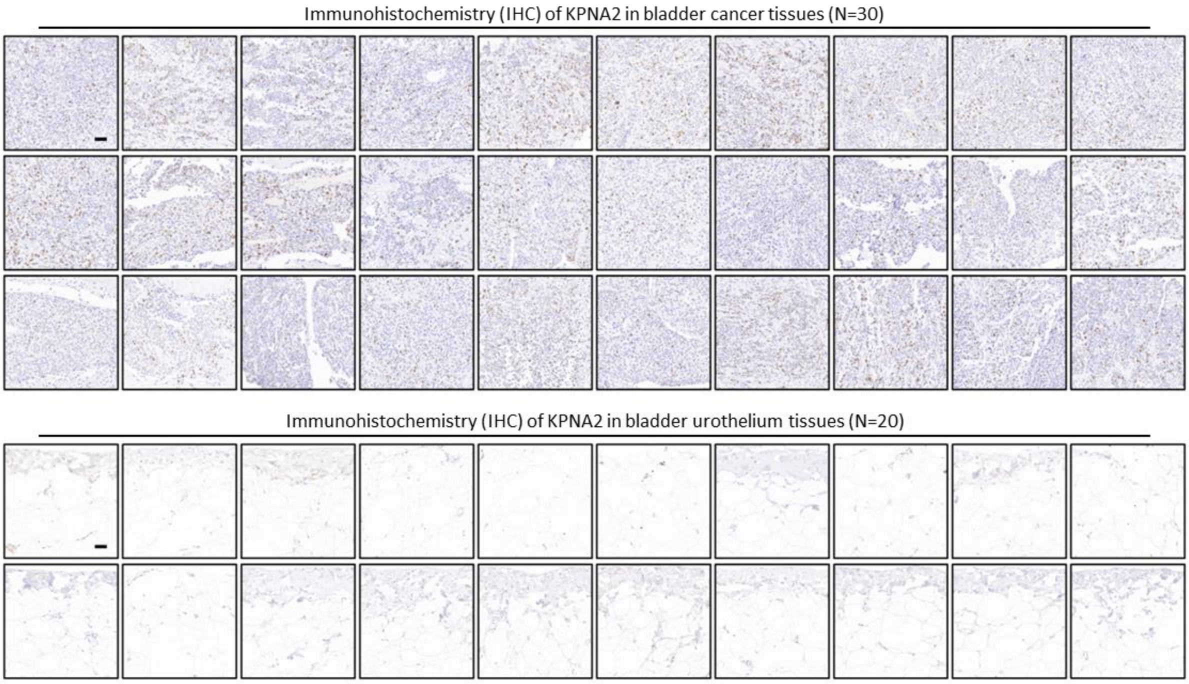

Fig. S18. KPNA2 immunohistochemical staining of bladder cancer tissues and bladder urothelium tissues. Scale bars: 50 μm.

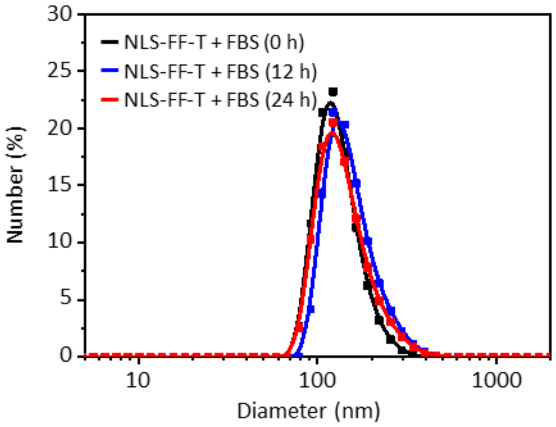

Fig. S19. Stability assay of **NLS-FF-T** in PBS containing 10% v/v fetal bovine serum (FBS) measured by DLS.

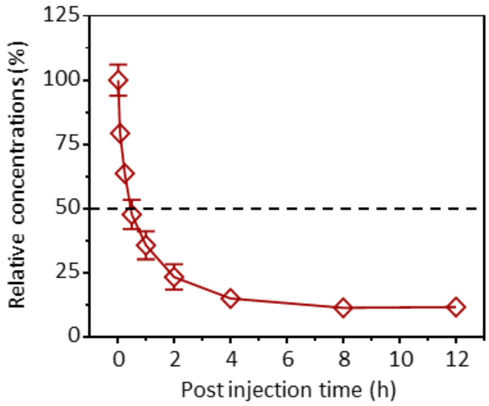

Fig. S20. The blood circulation curve of **NLS-FF-T** (50 μM) based on exponential curve fitting.

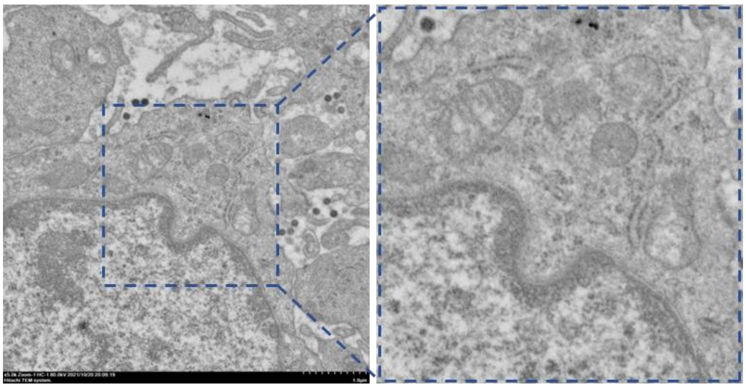

Fig. S21. Representative Bio-TEM images of T24 tumor tissues after treated with PBS.

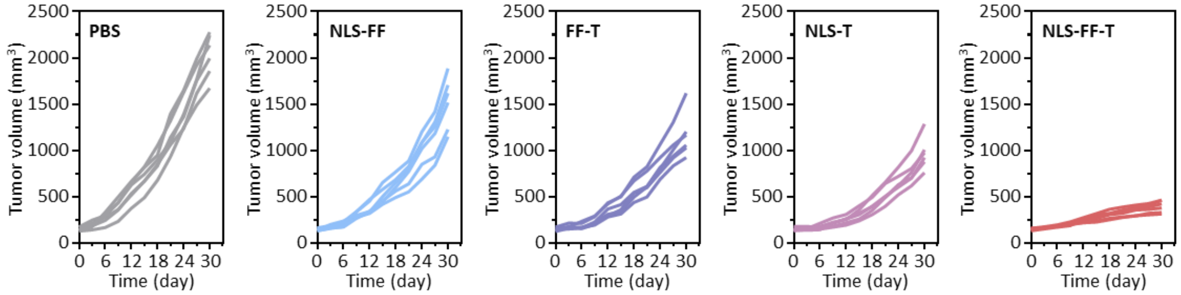

Fig. S22. The individual tumor growth curves of T24 xenograft mice after treated with PBS, NLS-FF, FF-T, NLS-T and **NLS-FF-T** (500 μM in 200 μL PBS) over 30 days.

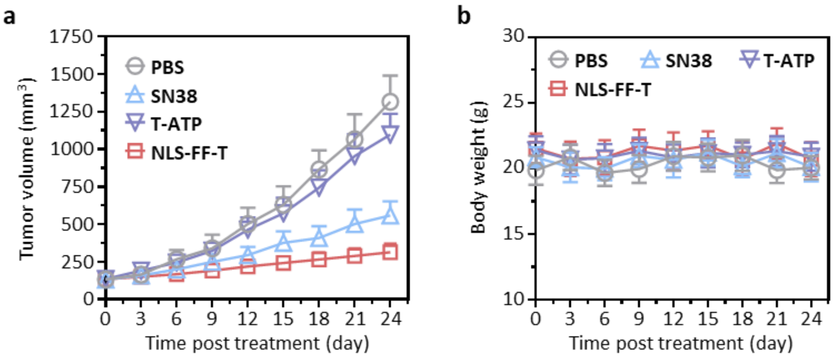

Fig. S23. (a) Average tumor growth curves of T24 xenograft mice after treated with PBS, SN38 (5 mg/kg), T-ATP and NLS-FF-T (500 μM in 200 μL PBS). **(b)** Body weights of T24 xenograft mice after different treatment.

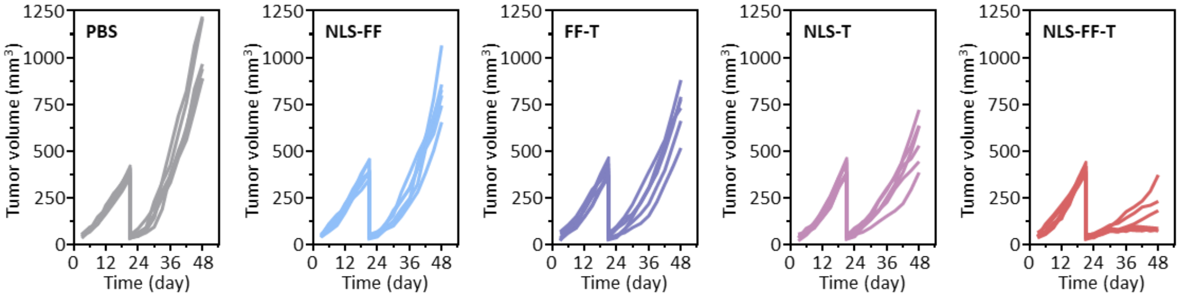

Fig. S24. The individual tumor growth curves of T24 xenograft mice after treated with PBS, NLS-FF, FF-T, NLS-T and **NLS-FF-T** (500 μM in 200 μL PBS) over 48 days.

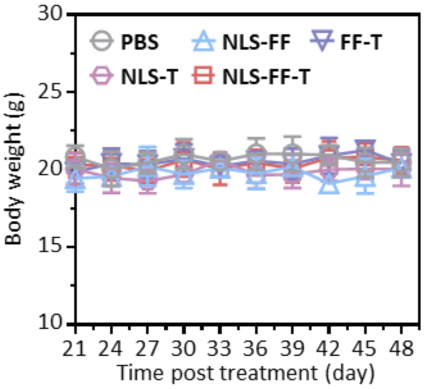

Fig. S25. Body weight changes of T24 xenograft mice after treated with PBS, NLS-FF, FF-T, NLS-T and **NLS-FF-T** (500 μM in 200 μL PBS).

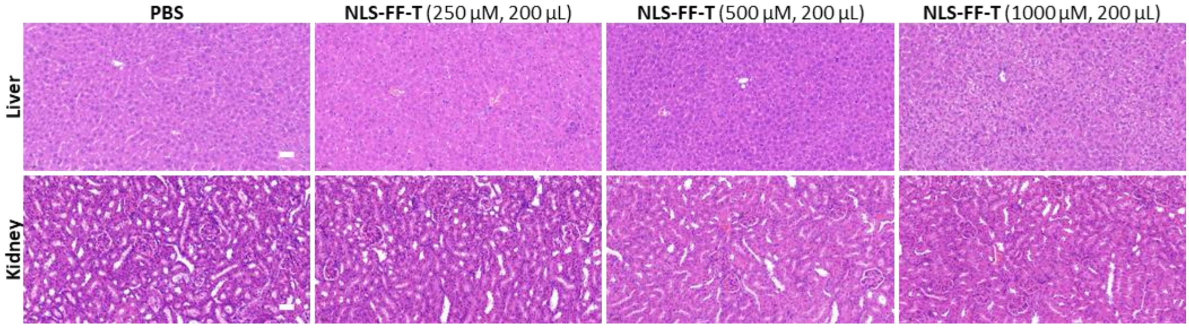

Fig. S26. Histology evaluation of the major organs (liver and kidney) by hematoxylin and eosin (H&E) after treatment with PBS and **NLS-FF-T** (1000 μM, 500 μM or 250 μM in 200 μL PBS) for six times (N = 4). Scale bar: 50 μm.

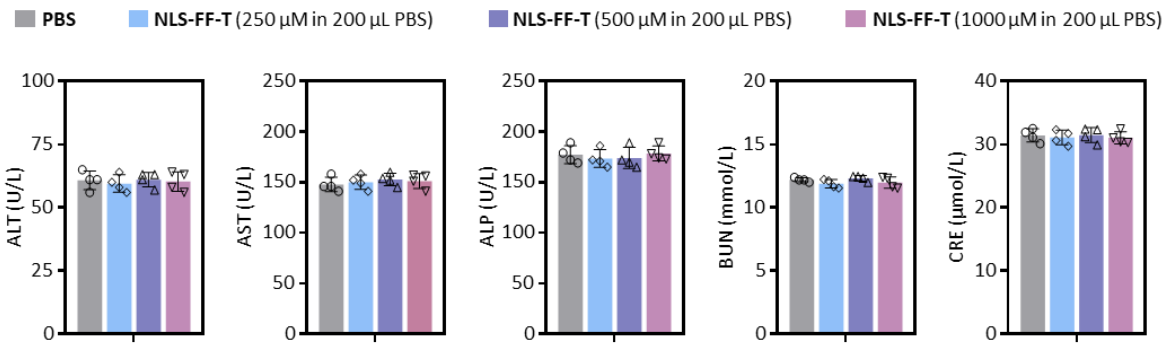

Fig. S27. Blood biochemistry data of the mice including alanine aminotransferase (ALT), aspartate aminotransferase (AST), alkaline phosphatase (ALP), total protein (TP), albumin (ALB), globulin (GLOB) blood urea nitrogen (BUN) and creatinine (CRE) after treatment with PBS and **NLS-FF-T** (1000 µM, 500 µM or 250 µM in 200 µL PBS) for six times (N = 4). Data were presented as mean ± SD.

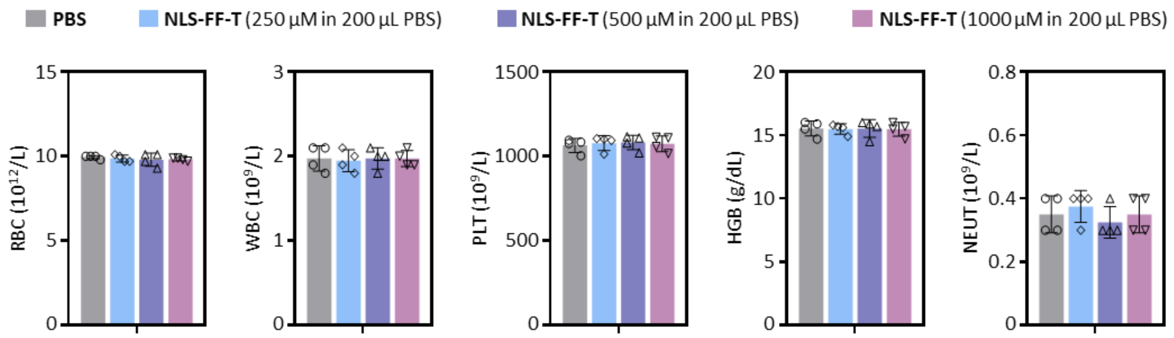

Fig. S28. Blood routine examination of the mice including red blood cell (RBC), white blood cell (WBC), platelets (PLT), hemoglobin (HGB) and neutrophil (NEUT) after treatment with PBS and **NLS-FF-T** (1000 μM, 500 μM or 250 μM in 200 μL PBS) for six times (N = 4). Data were presented as mean ± SD.
